# CCL5 paradoxically regulates glomerular injury by skewing macrophage polarization

**DOI:** 10.1172/jci.insight.173742

**Published:** 2025-09-23

**Authors:** Ika N. Kadariswantiningsih, Issei Okunaga, Kaho Yamasaki, Maulana A. Empitu, Hiroyuki Yamada, Shin-ichi Makino, Akitsu Hotta, Hideo Yagita, Masashi Aizawa, Ryo Koyama-Nasu, Motoko Y. Kimura, Narihito Tatsumoto, Katsuhiko Asanuma

**Affiliations:** 1 Department of Nephrology, Graduate School of Medicine, Chiba University, Chiba, Japan.; 2 Faculty of Medicine, Airlangga University, Surabaya, Indonesia.; 3 Department of Nephrology, Kyoto University Hospital, Kyoto, Japan.; 4 Department of Reprogramming Science, Center for iPS Cell Research and Application (CiRA), Kyoto University, Kyoto, Japan.; 5 Division of Cell Biology, Biomedical Research Center, Juntendo University Graduate School of Medicine, Tokyo, Japan.; 6 Department of Experimental Immunology, Graduate School of Medicine, Chiba University, Chiba, Japan.

**Keywords:** Inflammation, Nephrology, Chemokines, Chronic kidney disease, Macrophages

## Abstract

Glomerular inflammation and podocyte loss are the hallmarks of chronic kidney disease (CKD) progression. Understanding how podocytes and their microenvironment regulate inflammation is critical for developing effective therapies. In this study, we identified C-C chemokine ligand 5 (CCL5) as an inflammatory mediator elevated in injured podocytes, based on analyses of both human kidney biopsies and mouse models of CKD. We discovered that CCL5 exerts paradoxical effects in nephropathy; while it protects podocytes in vitro, it exacerbates glomerular injury in vivo. Recombinant CCL5 and podocyte-specific CCL5 overexpression promoted cell survival and reduced apoptosis in cultured podocytes. However, in adriamycin-induced nephropathy, CCL5 worsened glomerular injury, increasing proteinuria, glomerulosclerosis, and podocyte loss. Bone marrow (BM) transplantation experiments revealed that CCL5 in BM-derived cells — not kidney-resident cells — drove disease progression. CCL5 deficiency in BM-derived cells conferred protection by increasing reparative M2 macrophages, whereas endogenous CCL5 promoted M1 polarization, inhibited M2 differentiation, and triggered M2-to-M1 transition. These findings demonstrate that while CCL5 supports podocyte survival, its expression in BM-derived cells promotes inflammatory macrophage phenotypes and glomerular injury. The harmful immune effects of CCL5 in BM-derived cells outweigh its podocyte-protective role, highlighting the importance of cell-targeted strategies to mitigate kidney damage.

## Abstract 2

CCL5 protects kidney-filtering podocytes but worsens nephropathy by driving inflammatory macrophages from bone marrow, highlighting the need for cell-specific therapies in kidney disease.

## Introduction

Chronic kidney disease (CKD) is a major global health concern, contributing to significant morbidity and mortality in millions of individuals (1, 2). Despite its prevalence, the underlying mechanisms driving CKD progression remain incompletely understood, and effective therapeutic options remain limited. Identifying key molecular regulators of glomerular injury and inflammation is essential for developing targeted interventions to improve patient outcomes.

C-C chemokine ligand 5 (CCL5) is a protein implicated in various inflammatory diseases. Initially identified as a T cell–specific chemokine (3), CCL5 is now known to be expressed in multiple cell types, including macrophages, platelets, fibroblasts, endothelial, and epithelial cells (4–6). Due to its broad cellular expression, CCL5 plays a complex role in disease progression, contributing to both immune defense and inflammation-driven tissue damage (7, 8).

CCL5 expression is elevated in multiple kidney diseases, including acute renal failure, HIV nephropathy, transplant rejection, and focal segmental glomerulosclerosis (FSGS) (9). However, its role in CKD remains controversial. While some studies suggest that CCL5 inhibition attenuates glomerulonephritis, others report that CCL5 blockade worsens kidney injury, implying a potential protective role for CCL5 (10, 11). These conflicting findings highlight the need for further investigation into CCL5’s function in CKD pathogenesis.

In this study, we examined CCL5 expression in human glomerular diseases and in a CKD mouse model of adriamycin-induced (ADR-induced) nephropathy, which mimics FSGS — the primary cause of primary glomerular diseases leading to CKD (12, 13). We observed CCL5 localization in injured glomeruli of both humans and mice, where it colocalized with the podocyte marker synaptopodin. To investigate the functional impact of CCL5, we induced podocyte injury in vitro using wild-type (WT), CCL5-overexpressing (CCL5-OE), and *Ccl5* knockout (*Ccl5*-KO) podocytes. Additionally, we examined ADR-induced nephropathy in vivo using *Ccl5*-KO and bone marrow transplant (BMT) models.

By combining in vitro and in vivo studies, we explored the dual role of CCL5 in podocytes and macrophages, two key cell types involved in ADR-induced nephropathy pathogenesis. Our findings provide insights into the complex role of CCL5 in CKD and may help resolve previous discrepancies regarding its contribution to glomerular injury and repair.

## Results

### CCL5 is expressed in podocytes and upregulated in human and mouse glomerular injury.

**Figure 1:**
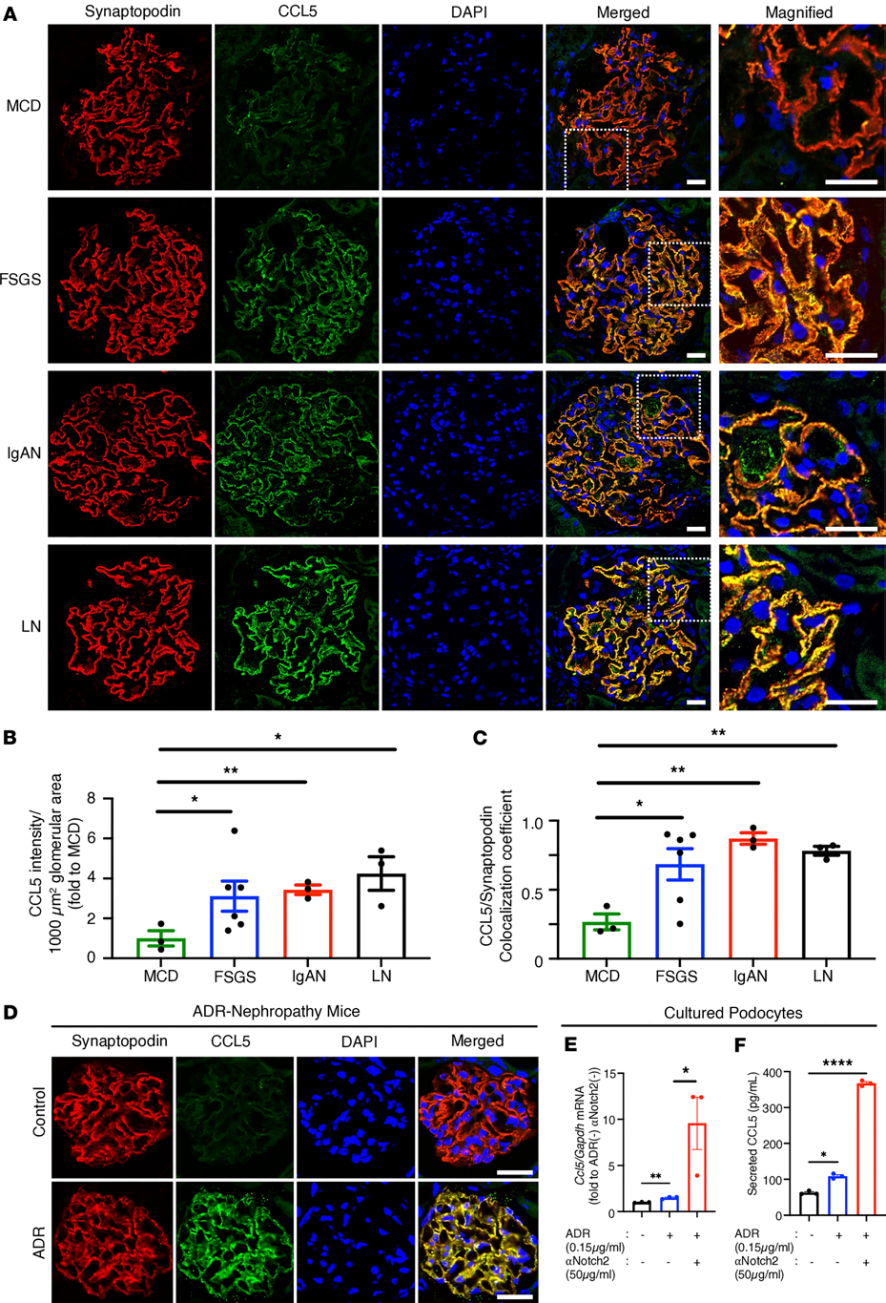
CCL5 expression in podocytes of human glomerular diseases and ADR-induced nephropathy mice. (**A**) Representative glomerular immunofluorescence images of synaptopodin (red) and CCL5 (green) in human glomerular diseases of MCD (*n* = 3), FSGS (*n* = 6), IgAN (*n* = 3), and LN (*n* = 3). (**B** and **C**) Quantification of CCL5 immunofluorescence intensity and colocalization of synaptopodin and CCL5 in human glomerular diseases. (**D**) Glomerular immunofluorescence images of synaptopodin (red) and CCL5 (green) in control or WT mice 3 days after ADR injection. (**E**) Relative mRNA expression of *Ccl5* in cultured podocytes that were untreated (*n* = 3) or stimulated with 0.15 μg/mL ADR (*n* = 3) or a combination of 0.15 μg/mL ADR and 50 μg/mL αNotch2 (*n* = 3) for 6 hours. Measured values were normalized to *Gapdh* and calculated by the ΔΔCt method. (**F**) The concentration of CCL5 secreted by cultured podocytes that were untreated (*n* = 3) or stimulated with 0.15 μg/mL ADR (*n* = 3) or a combination of 0.15 μg/mL ADR and 50 μg/mL αNotch2 (*n* = 3) for 12 hours. All data are expressed as mean ± SEM. Unpaired 2-tailed *t* tests; 1-way ANOVA; Dunnett’s multiple comparison test for **B**, **C**, and **F**; and Tukey’s multiple comparison test for **E** were performed to calculate the P values. *P < 0.05; **P < 0.01; ****P < 0.0001. Scale bars: 20 μm.

To investigate the role of CCL5 in glomerular injury, we analyzed its expression in human kidney biopsies and ADR-induced nephropathy mice. Kidney biopsy samples from patients with minimal change disease (MCD), characterized by minor glomerular abnormalities, mild clinical symptoms, steroid responsiveness, and no relapse, were used as controls. In patients with FSGS, IgA nephropathy (IgAN), and lupus nephritis (LN), CCL5 was highly expressed in the glomeruli and showed a capillary pattern that colocalized with synaptopodin, a podocyte-specific marker (Figure 1A). Quantitative analysis revealed significantly higher CCL5 intensity in FSGS, IgAN, and LN compared with MCD (Figure 1B), and the degree of CCL5 and synaptopodin colocalization was also increased (Figure 1C), suggesting upregulation of CCL5 in glomerular disease. To provide an overview of the study population, we have included a table summarizing the clinical characteristics of the biopsy samples (Supplemental Table 1; supplemental material available online with this article; https://doi.org/10.1172/jci.insight.173742DS1).

To further contextualize CCL5 expression in diseased glomeruli, we examined CCL5 staining in normal human kidney samples. Immunofluorescence analysis revealed that CCL5 expression was minimal to undetectable in normal glomeruli (Supplemental Figure 1), suggesting that CCL5 is expressed at low levels under physiological conditions but becomes upregulated in response to glomerular injury. These findings provide a baseline reference for CCL5 expression in healthy kidneys, further highlighting its induction in glomerular diseases.

A similar pattern was observed in ADR-induced nephropathy mice, where CCL5 was upregulated in podocytes 3 days after ADR injection, showing a capillary pattern colocalized with synaptopodin (Figure 1D). To ensure antibody specificity, we performed immunofluorescent staining of CCL5 in ADR-injected *Ccl5*-KO mice, confirming the expected absence of CCL5 signal (Supplemental Figure 2). These results suggested that CCL5 expression is upregulated in podocytes in response to glomerular injury in both human disease and ADR nephropathy models.

Previous studies have linked increased C-C motif proteins, such as CCL5, to the activation of Notch receptors (14, 15), and Notch2 signaling has been shown to protect podocytes and glomeruli from ADR-induced injury (16). Therefore, we hypothesized that ADR-induced CCL5 upregulation in podocytes might follow a Notch2 activation pattern.

To evaluate this, we treated murine cultured podocytes with ADR or ADR plus an anti-Notch2 agonist antibody (αNotch2) and measured *Ccl5* mRNA expression (quantitative real-time PCR, qPCR) and protein secretion (ELISA) (Figure 1, E and F). CCL5 expression significantly increased following ADR treatment and was further enhanced by αNotch2 stimulation (Figure 1, E and F). This pattern closely mirrored Notch2 activation in podocytes, as previously described by Tanaka et al. (16), suggesting a potential link between ADR-induced CCL5 expression and Notch2 signaling.

To further confirm that podocytes express CCL5, we performed immunofluorescent staining of CCL5 and synaptopodin in WT cultured podocytes. CCL5 was detected intracellularly and colocalized with synaptopodin, demonstrating that CCL5 is expressed in podocytes under baseline conditions, even in the absence of injury (Supplemental Figure 3). These findings align with our mRNA expression and protein secretion analyses, further validating that podocytes are a key source of CCL5 in the glomerulus.

Since CCL5 can signal through multiple GPCRs, we examined podocyte mRNA expression of *Ccr1*, *Ccr3*, *Ccr5*, and *Gpr75*, which are all known CCL5 receptors (17, 18). Among these, only mRNA expression of *Gpr75* was detected in cultured podocytes (Supplemental Figure 4), suggesting that CCL5 primarily signals via GPR75 in podocytes rather than through CCR1, CCR3, or CCR5.

Together, these findings demonstrate that CCL5 is upregulated in podocytes in both human and mouse models of glomerular injury, with its expression in ADR-induced nephropathy following a pattern similar to Notch2 activation. Moreover, the data suggest that CCL5 likely signals via GPR75 in podocytes during glomerular injury, highlighting its potential role in disease progression.

### Exogenous and endogenous CCL5 protects podocytes from apoptosis.

Since CCL5 is induced in ADR-treated podocytes, similar to Notch2 activation, we investigated whether CCL5 plays a protective role in ADR-induced nephropathy by assessing podocyte apoptosis. We included the full gating strategy used in the Annexin V/7-AAD analysis for evaluating apoptosis (Supplemental Figure 5).

**Figure 2:**
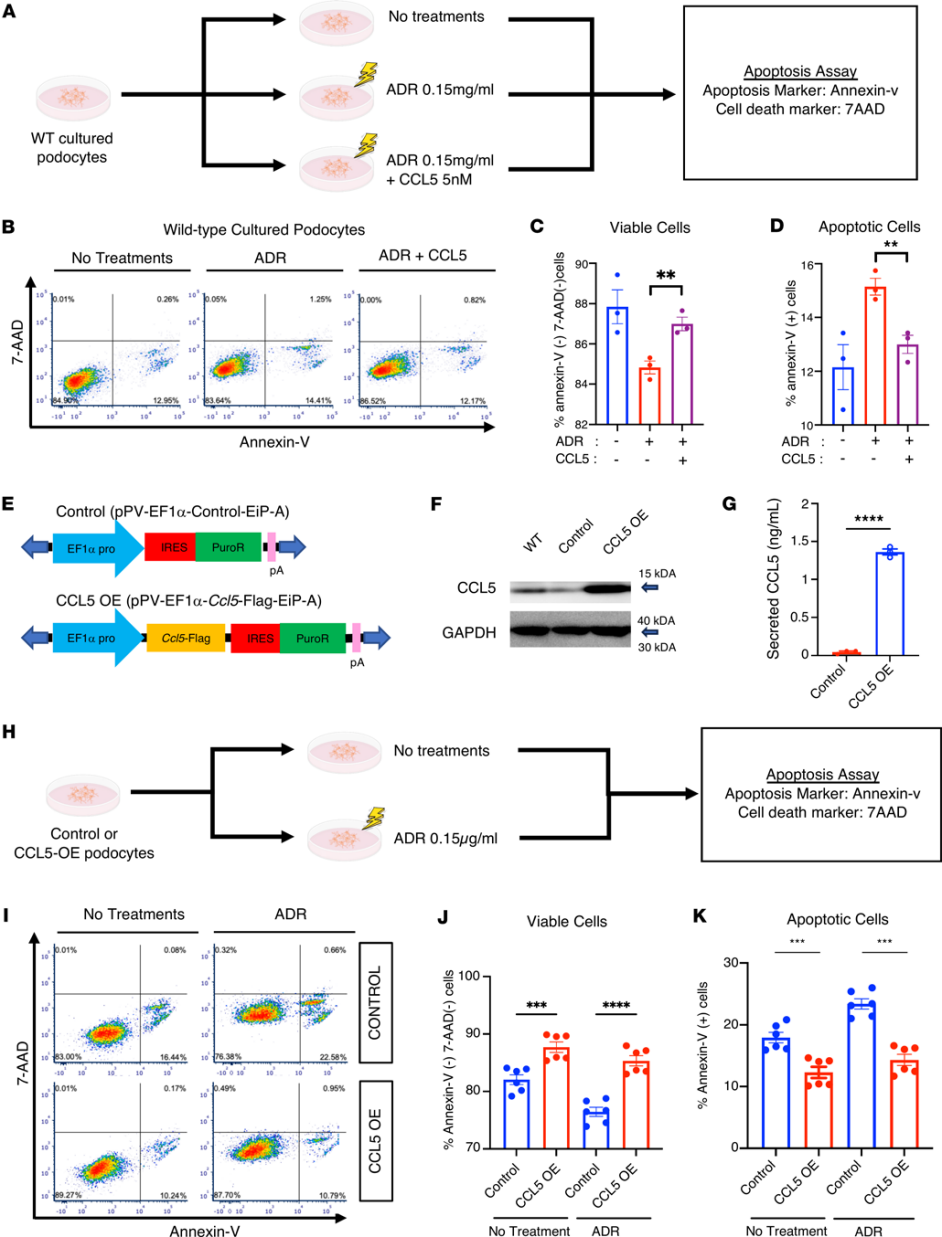
The effect of exogenous and endogenous CCL5 in the apoptosis of podocytes. (**A**) Experimental scheme of the apoptosis assay in WT murine cultured podocytes that were untreated (*n* = 3) or treated with 0.15 μg/mL ADR (*n* = 3) or a combination of 0.15 μg/mL ADR and 5 nM CCL5 (*n* = 3). (**B**) Representative result of the flow cytometry–based apoptosis assay, measuring death cell marker 7-AAD and apoptosis marker Annexin V in untreated, ADR-treated, and ADR+CCL5-treated WT cultured podocytes. (**C** and **D**) Percentage of viable and apoptotic cells in untreated, ADR-treated, and ADR+CCL5-treated cultured podocytes. (**E**) Schematic design of transfected plasmid DNA of control and CCL5-OE cultured podocytes. (**F**) Western blot analysis of WT, control, and CCL5-OE cultured podocytes. (**G**) Concentration of CCL5 secreted by control and CCL5-OE cultured podocytes. (**H**) Experimental scheme of the apoptosis assay in the untreated control (*n* = 6), untreated CCL5-OE (*n* = 6), ADR-treated control (*n* = 6), and ADR-treated CCL5-OE cultured podocytes (*n* = 6). (**I**) Representative flow cytometry–based apoptosis assay result, measuring death cell marker 7-AAD and apoptosis marker Annexin V in untreated and ADR-treated control and CCL5-OE cultured podocytes. (**J** and **K**) Percentage of viable and apoptotic cells in untreated control, untreated CCL5-OE, ADR-treated control, and ADR-treated CCL5-OE cultured podocytes. All data are expressed as mean ± SEM. Unpaired 2-tailed *t* tests, 1-way ANOVA tests, and Tukey’s multiple comparison test for panels **C**, **D**, **J**, and **K** were performed to calculate the *P* values. **P < 0.01; ***P < 0.001; ****P < 0.0001. IRES, internal ribosome entry site; Puro, puromycin.

We treated WT cultured podocytes with ADR alone or a combination of ADR and exogenous CCL5, and then analyzed the apoptosis markers Annexin V and 7-AAD (Figure 2A). As expected, ADR treatment significantly increased apoptosis (Figure 2, B–D). However, exogenous CCL5 treatment enhanced podocyte viability and reduced apoptosis in ADR-treated podocytes (Figure 2, C and D), suggesting that CCL5 protects podocytes from ADR-induced apoptosis.

To assess the effect of endogenous CCL5, we generated CCL5-OE podocytes using the piggyBac transposon system (Figure 2E). Western blotting and ELISA confirmed that CCL5-OE podocytes expressed and secreted a significantly higher level of CCL5 protein than control podocytes (Figure 2, F and G).

We then conducted an apoptosis assay comparing CCL5-OE podocytes and control podocytes, both under normal conditions and ADR treatment (Figure 2, H and I). CCL5 overexpression significantly increased podocyte viability and reduced apoptosis, even in ADR-treated cells (Figure 2, J and K). Early apoptotic cells (Annexin V^+^7-AAD^–^) were also significantly reduced in CCL5-OE podocytes under both conditions, further supporting the protective role of CCL5 (Supplemental Figure 6).

These findings demonstrate that both exogenous and endogenous CCL5 protect podocytes from ADR-induced apoptosis, reinforcing its potential role in maintaining podocyte survival during glomerular injury.

### CCL5 deficiency increases ADR-induced apoptosis in cultured podocytes.

**Figure 3:**
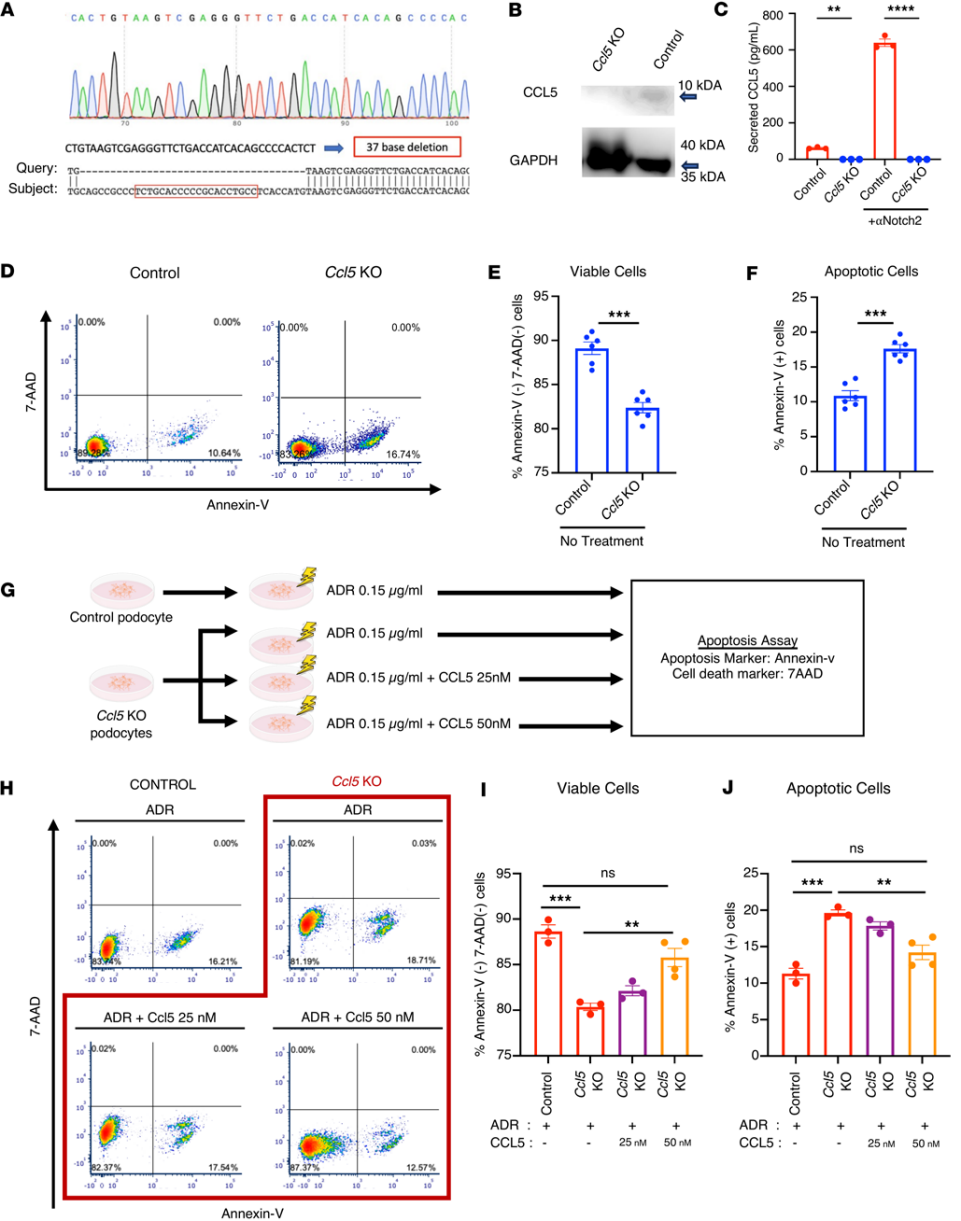
The effect of CCL5 deficiency in ADR-treated cultured podocytes. (**A**) Sanger sequence analysis of *Ccl5*-KO podocytes. Red box indicates target site of sgRNA. (**B**) Western blot analysis of control and *Ccl5*-KO cultured podocytes. (**C**) The concentration of CCL5 secreted by control and *Ccl5*-KO cultured podocytes under untreated conditions or stimulation with 50 μg/mL αNotch2. (**D**) Representative result of the flow cytometry–based apoptosis assay measuring the death cell marker 7-AAD and the apoptosis marker Annexin V in untreated control and *Ccl5*-KO cultured podocytes. (**E** and **F**) Percentage of viable and apoptotic cells in untreated control and *Ccl5*-KO cultured podocytes. (**G**) Experimental scheme of the apoptosis assay in control and *Ccl5-*KO cultured podocytes. Apoptosis assay was performed in the untreated control (*n* = 6), untreated *Ccl5*-KO (*n* = 6), ADR-treated control (*n* = 3), ADR-treated *Ccl5*-KO (*n* = 3), and in *Ccl5*-KO cultured podocytes treated with a combination of ADR and 25 nM CCL5 (*n* = 3) or a combination of ADR and 50 nM CCL5 (*n* = 4). (**H**) Representative result of the flow cytometry–based apoptosis assay measuring the death cell marker 7-AAD and the apoptosis marker Annexin V in ADR-treated control, ADR-treated *Ccl5*-KO cultured podocytes, and in *Ccl5*-KO cultured podocytes treated with a combination of ADR and CCL5. (**I** and **J**) Percentage of viable and apoptotic cells in ADR-treated control, ADR-treated *Ccl5*-KO cultured podocytes, and *Ccl5*-KO cultured podocytes treated with a combination of ADR and CCL5. All data are expressed as mean ± SEM. Unpaired 2-tailed *t* tests, 1-way ANOVA, and Tukey’s multiple comparison test for panels **I** and **J** were performed to calculate the *P* values. **P < 0.01; ***P < 0.001; ****P < 0.0001. NS, not significant.

To investigate the role of CCL5 in podocyte survival, we generated *Ccl5*-KO podocytes using the clustered regular interspaced short palindromic repeats (CRISPR)/Cas9 gene editing system. To assess the specificity of CRISPR editing, we analyzed predicted off-target sites using Cas-OFFinder (Supplemental Table 2). Here we found that none of the predicted off-targets were located in genes directly related to kidney function, suggesting a low likelihood of unintended gene disruptions influencing our findings at the podocyte level. Sequencing confirmed a 37–base pair deletion in the target sequence (Figure 3A); Western blot and ELISA validated that *Ccl5*-KO podocytes did not express or secrete CCL5 protein (Figure 3, B and C).

We then evaluated the impact of CCL5 deficiency on podocyte viability and apoptosis. Under physiological conditions, *Ccl5*-KO podocytes exhibited reduced viability and increased spontaneous apoptosis (Figure 3, D–F), indicating that CCL5 is necessary for maintaining podocyte survival.

To further evaluate this effect under stress conditions, we treated *Ccl5*-KO and control podocytes with ADR and measured apoptosis (Figure 3, G and H). Loss of CCL5 exacerbated ADR-induced apoptosis and further reduced podocyte viability (Figure 3, I and J). However, when *Ccl5*-KO podocytes were treated with both ADR and exogenous CCL5, apoptosis was significantly reduced, and podocyte viability was restored to levels comparable to control podocytes (Figure 3, I and J).

We also evaluated early apoptotic events defined as Annexin V^+^7-AAD^–^ cells. The percentage of early apoptotic cells was significantly higher in *Ccl5*-KO podocytes with ADR compared with control treated with ADR. Treatment with exogenous CCL5 significantly decreased early apoptosis in *Ccl5*-KO podocytes, restoring it to levels comparable to control cells (Supplemental Figure 7). These findings demonstrate that CCL5 plays a crucial role in protecting podocytes from both spontaneous and ADR-induced apoptosis, as its deficiency increases cell death while exogenous CCL5 restores podocyte survival.

### CCL5 deficiency ameliorates ADR-induced nephropathy in mice.

**Figure 4:**
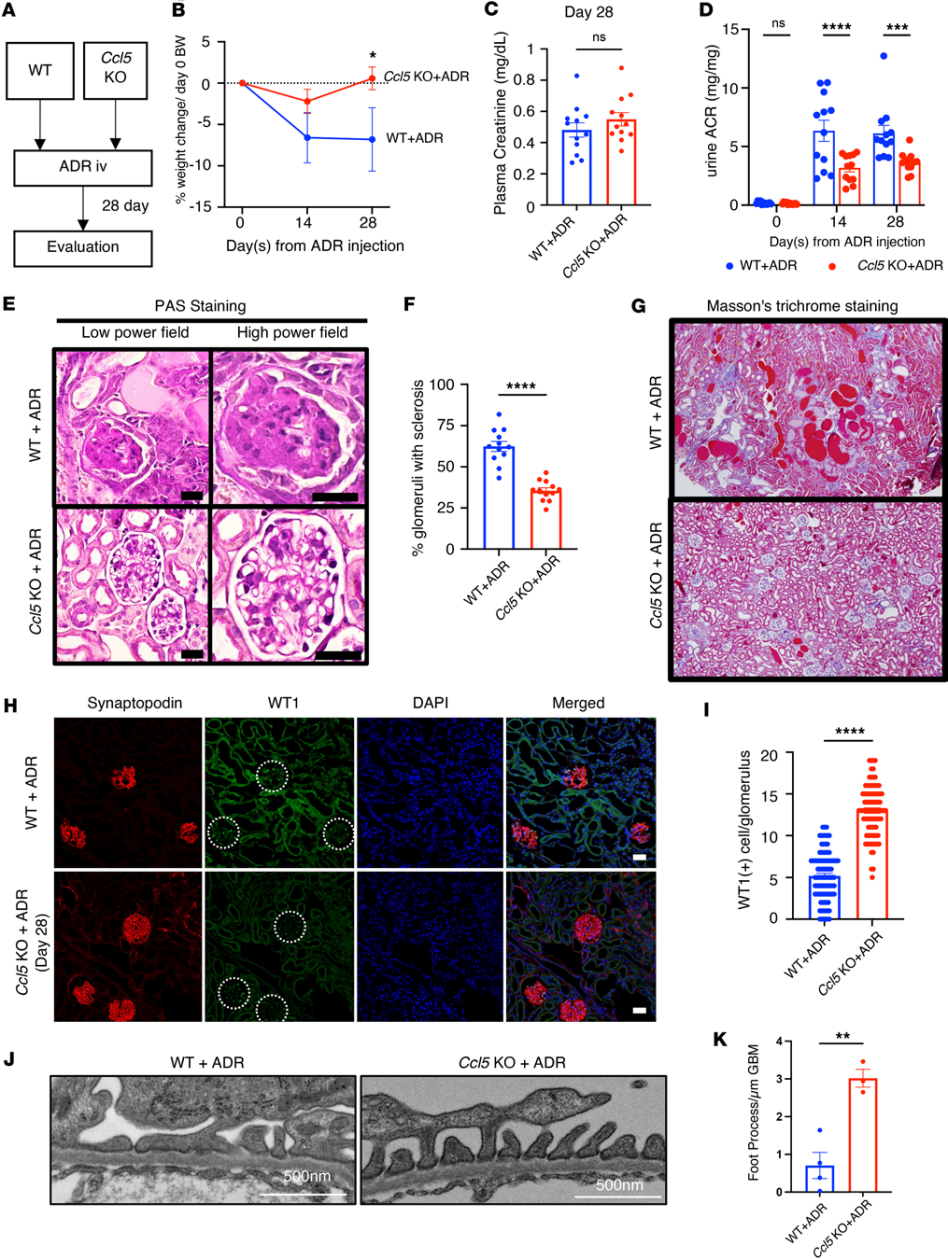
The effect of CCL5 deficiency in ADR-induced nephropathy mice. (**A**) ADR-induced nephrosis was induced in *Ccl5*-KO mice (*n* = 12) and their WT littermates (*n* = 12). (**B**) Percentage of body weight change from the initial body weight after ADR injection. (**C**) Plasma creatinine on day 28 after ADR injection. (**D**) Urine ACR of *Ccl5*-KO and WT mice treated with ADR. (**E**) Representative PAS staining image of kidney, sacrificed on day 28 after ADR injection. Scale bars: 30 μm. (**F**) Quantification of sclerotic glomeruli per total glomeruli. (**G**) Representative Masson’s trichrome staining of kidney, sacrificed on day 28 after ADR injection. (**H**) Representative confocal microscopy analysis of WT-1 and synaptopodin immunofluorescent staining in ADR-treated WT and *Ccl5*-KO mice. Scale bars: 30 μm. (**I**) Quantification of the number of cells with positive WT-1 staining per glomerulus from *Ccl5* -KO and WT mice treated with ADR (7–8 glomeruli counted per mouse), analyzed by confocal microscopy of immunofluorescent staining. (**J**) Representative electron microscopy analysis of kidney, sacrificed on day 28 after ADR injection. Scale bars: 500 nm. (**K**) Quantification of the number of foot process per μm of glomerular basement membrane from *Ccl5*-KO and WT mice treated with ADR. All data are presented as mean ± SEM. Unpaired 2-tailed *t* tests were performed to calculate the *P* values. *P < 0.05, **P < 0.01, ***P < 0.001, ****P < 0.0001. Urine ACR, urine albumin-to-creatinine ratio; NS, not significant.

To evaluate the in vivo role of CCL5 in ADR-induced nephropathy, we injected WT and *Ccl5*-KO mice with ADR and then compared the glomerular pathology (Figure 4A). After ADR injection, WT mice showed more severe body weight loss than *Ccl5*-KO mice (Figure 4B), suggesting a more severe systemic response. Although plasma creatinine levels remained similar between groups 28 days after injection (Figure 4C), *Ccl5*-KO mice showed significantly milder albuminuria than WT mice (Figure 4D).

Histological analysis further revealed that *Ccl5*-KO mice had fewer sclerotic glomeruli than WT mice, indicating reduced kidney injury (Figure 4, E and F). Additionally, Masson’s trichrome staining confirmed less glomerular fibrosis in *Ccl5*-KO mice (Figure 4G), consistent with milder structural injury in the absence of CCL5.

To evaluate podocyte loss, we performed immunofluorescent staining for Wilms tumor protein 1 (WT-1) and synaptopodin, markers of podocyte nuclei. *Ccl5*-KO mice retained more WT-1^+^ podocytes per glomerulus than WT mice (Figure 4, H and I), demonstrating that CCL5 deficiency reduced podocyte loss in ADR-induced nephropathy.

To further investigate the structural integrity of podocytes, we analyzed electron microscopy images of glomeruli. *Ccl5*-KO mice exhibited a higher number of podocyte foot processes per area of glomerular basement membrane (GBM) than WT mice following ADR treatment, suggesting that CCL5 deficiency helps preserve podocyte morphology and prevents foot process effacement (Figure 4, J and K).

Together, these findings indicate that CCL5 deficiency provides a protective effect in ADR-induced nephropathy by reducing albuminuria, glomerular sclerosis, podocyte loss, and foot process effacement, suggesting that CCL5 contributes to the progression of glomerular injury.

### CCL5 deficiency in BM-derived cells ameliorates ADR-induced nephropathy.

While in vitro findings demonstrated that CCL5 protects podocytes from apoptosis, in vivo results revealed that CCL5 exacerbated ADR-induced nephropathy in mice. Since our model used global *Ccl5*-KO mice, the observed effects could stem from CCL5 loss in both podocytes and immune cells, particularly BM-derived cells, which may play a dominant role in disease progression.

These immune cells might potentially be involved in modulating the outcome of ADR-induced nephropathy. Therefore, we hypothesized that the discrepancy between in vitro and in vivo findings might be attributable to the influence of CCL5 in BM-derived cells. The deleterious effect of CCL5 in the glomeruli could be caused by the CCL5 effect on BM-derived cells that overpowers its effect on podocytes.

**Figure 5:**
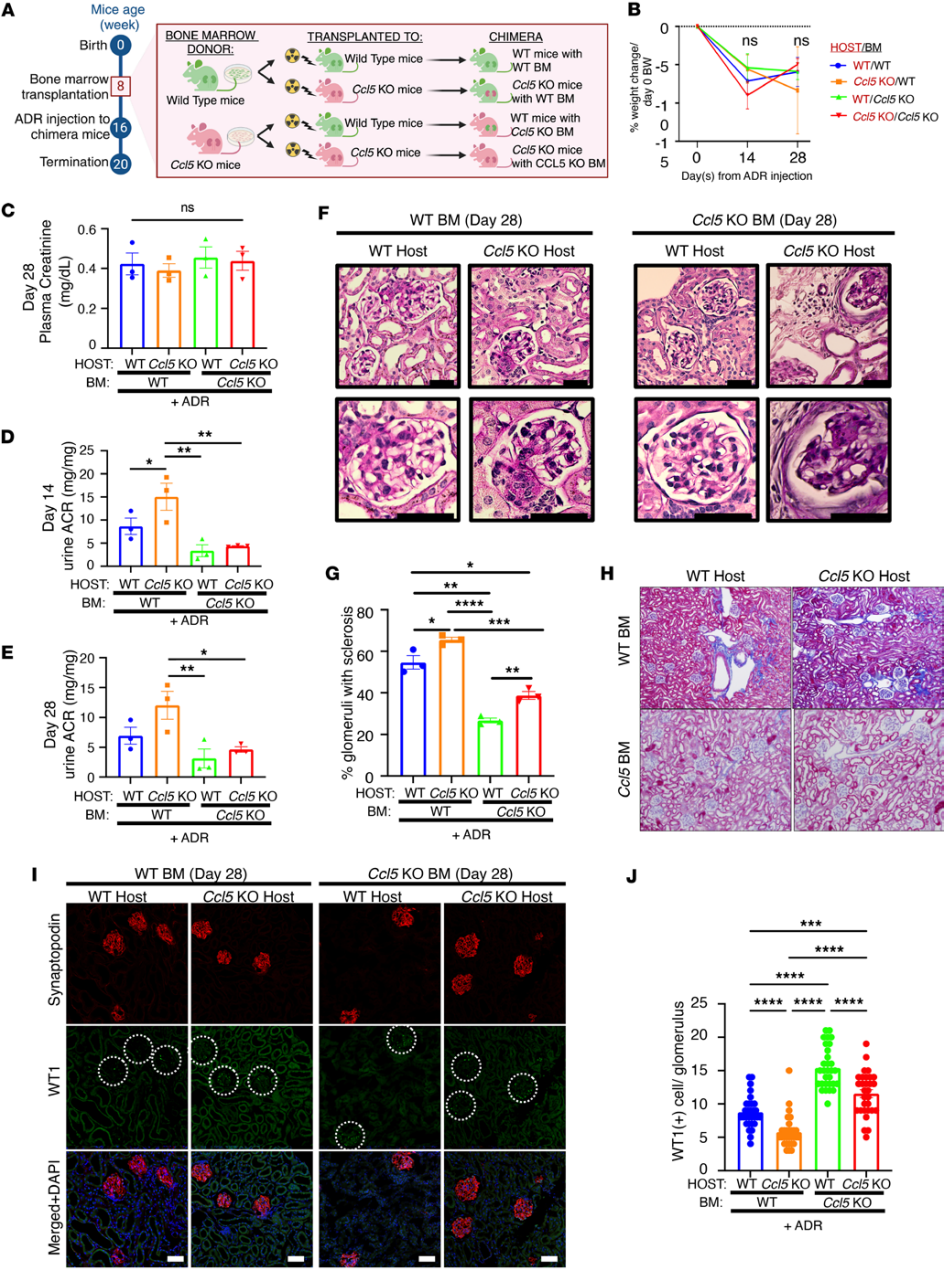
The effect of CCL5 deficiency in BM-derived cells on ADR-induced nephropathy. (**A**) Experimental diagram of the establishment of ADR-induced nephropathy in BMT mice. (**B**) Percentage of body weight change from the initial body weight after ADR injection. (**C**) Plasma creatinine on day 28 after ADR injection. (**D** and **E**) Urine ACR of BMT mice treated with ADR on day 14 and 28 after ADR injection. (**F**) Representative PAS staining image of the kidney of ADR-injected BMT mice, sacrificed on day 28 after ADR injection. Scale bars: 50 μm. (**G**) Quantification of sclerotic glomeruli per total glomeruli. (**H**) Representative Masson’s trichrome staining of kidney, sacrificed on day 28 after ADR injection. (**I**) Representative confocal microscopy analysis of WT-1 and synaptopodin immunofluorescent staining in ADR-treated BMT mice. Scale bars: 50 μm. (**J**) Quantification of the number of cells with positive WT-1 staining per glomerulus from ADR-treated BMT mice (10 glomeruli counted per mouse), analyzed by confocal microscopy of immunofluorescent staining. All data are represented as mean ± SEM. One-way ANOVA tests were performed to calculate the *P* values. All panels with multiple comparison bar graphs are using 1-way ANOVA. Tukey’s multiple comparison test for panels **C** and **G**; Uncorrected Fisher’s LSD for **D**, **E**, and **J**. *P < 0.05; **P < 0.01; ***P < 0.001; ****P < 0.0001. Urine ACR, urine albumin-to-creatinine ratio; NS, not significant.

To distinguish the effects of CCL5 in BM-derived cells versus kidney-resident cells, we performed BMT experiments. WT or *Ccl5*-KO BM was transplanted into WT or *Ccl5*-KO host mice, generating 4 experimental groups (Figure 5A). Eight weeks after BMT, we verified the successful engraftment of donor BM in host mice (Supplemental Figure 8) before inducing ADR nephropathy. Glomerular pathology was analyzed on day 28 after ADR injection to determine the contribution of CCL5 in different cell populations.

There were no significant differences in body weight loss or plasma creatinine levels among the 4 groups (Figure 5, B and C). However, mice that received *Ccl5*-KO BM exhibited significantly less albuminuria, fewer sclerotic glomeruli, and a greater number of WT-1^+^ podocytes per glomerulus, regardless of host type, compared with those that received WT BM (Figure 5, D–I). Masson’s trichrome staining further confirmed that mice transplanted with *Ccl5*-KO BM had reduced glomerular fibrosis compared with those with WT BM (Figure 5H), supporting the notion that CCL5 in BM-derived cells drives fibrotic and structural injury in the glomeruli. These findings indicate that CCL5 deficiency in BM-derived cells protects against ADR-induced nephropathy.

In contrast, *Ccl5*-KO host mice exhibited more severe albuminuria, increased glomerulosclerosis, and fewer WT-1^+^ podocytes per glomerulus, regardless of BM donor type (Figure 5, D and F–I). These results suggest that CCL5 deficiency in kidney-resident cells worsens ADR-induced nephropathy, indicating a potentially protective function of CCL5 within the glomerular microenvironment.

Comparing nephropathy severity across groups, mice with CCL5 expression restricted to kidney-resident cells exhibited the mildest nephropathy, suggesting that CCL5 in kidney-resident cells may play a protective role against ADR-induced nephropathy. In contrast, mice with CCL5 expression only in BM-derived cells developed the most severe nephropathy, highlighting BM-derived CCL5 as the primary driver of ADR-induced glomerular injury (Figure 5, D–J).

These findings indicated that the detrimental effects of CCL5 in ADR-induced nephropathy are largely driven by its presence in BM-derived cells. This deleterious influence outweighs the potentially protective role of CCL5 in kidney-resident cells, highlighting BM-derived CCL5 as a dominant contributor to glomerular injury.

### CCL5 in BM-derived cells limits glomerular M2 macrophage accumulation in ADR nephropathy.

To determine which BM-derived cell type contributes to the deleterious effects of CCL5 in ADR-induced nephropathy, we investigated its role in macrophage polarization. Previous studies have shown that CCL5 is strongly associated with macrophages (19, 20). CCL5 plays an essential role in macrophage survival and monocyte-macrophage trafficking into inflamed sites (21, 22). In response to kidney injury, chemokines induce naive monocyte infiltration, which differentiate into M0 macrophages and subsequently polarize into proinflammatory M1 macrophages or antiinflammatory M2 macrophages (23). While M1 macrophages drive inflammation, M2 macrophages promote tissue repair (24), and the balance between these phenotypes is critical for organ recovery (25). Given CCL5’s established role in macrophage regulation, we hypothesized that CCL5 exacerbates ADR-induced nephropathy by skewing macrophage polarization toward the proinflammatory M1 phenotype, thereby reducing reparative M2 macrophages.

**Figure 6:**
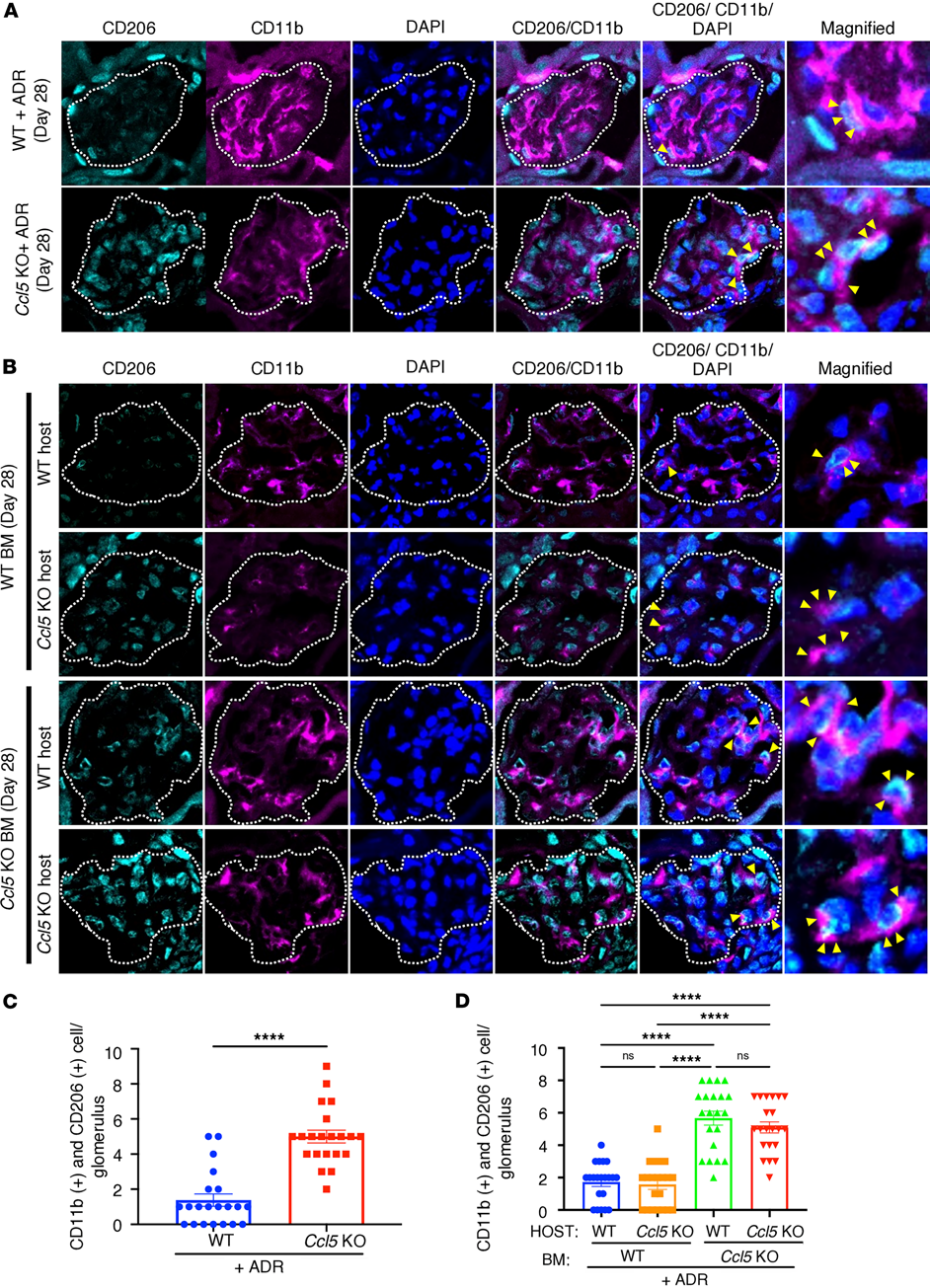
CCL5 in BM-derived cells limits glomerular M2 macrophage accumulation in ADR-induced nephropathy. ADR-injected mouse kidneys were stained for CD11b (pan macrophage marker) and CD206 (M2 macrophage marker) to evaluate M2 macrophages in the glomeruli. The dashed white line circle represents the glomerulus. (**A**) Representative image of M2 macrophage infiltration in the glomeruli of WT (*n* = 3) and *Ccl5*-KO mice (*n* = 3) treated after ADR injection. (**B**) Representative images of M2 macrophage in the glomeruli of ADR-treated BMT mice (*n* = 3 for each group). (**C**) Quantification of M2 macrophage per glomeruli in WT and *Ccl5*-KO mice treated with ADR (7 glomeruli counted per mouse). (**D**) Quantification of M2 macrophages per glomeruli in ADR-treated BMT mice (7 glomeruli counted per mouse). All data are represented as mean ± SEM. Unpaired 2-tailed *t* tests and 1-way ANOVA with uncorrected Fisher’s LSD were performed to calculate the *P* values. NS, not significant. ****P < 0.0001.

To test this hypothesis, we injected WT and *Ccl5*-KO mice with ADR and examined M2 macrophage presence in glomeruli using CD11b (pan-macrophage marker) and CD206 (M2 macrophage marker, encoded by *Mrc1*) staining. Immunofluorescence analysis on day 28 after ADR injection revealed CD11b and CD206 colocalization in the glomeruli of both WT and *Ccl5*-KO mice (Figure 6A), as well as in BMT mice (Figure 6B), confirming the presence of glomerular M2 macrophages in ADR nephropathy.

Quantitative analysis showed that 28 days after ADR injection, WT mice had a lower number of glomerular M2 macrophages compared with *Ccl5*-KO mice (Figure 6C). Similarly, in BMT mice, host mice with WT BM exhibited fewer glomerular M2 macrophages than those transplanted with *Ccl5*-KO BM (Figure 6D). These findings indicated that CCL5 in the BM-derived cells reduced the glomerular M2 macrophage population, potentially limiting the reparative response in ADR-induced nephropathy.

### CCL5 in macrophages exacerbates ADR-induced nephropathy by limiting glomerular M2 macrophage accumulation.

**Figure 7:**
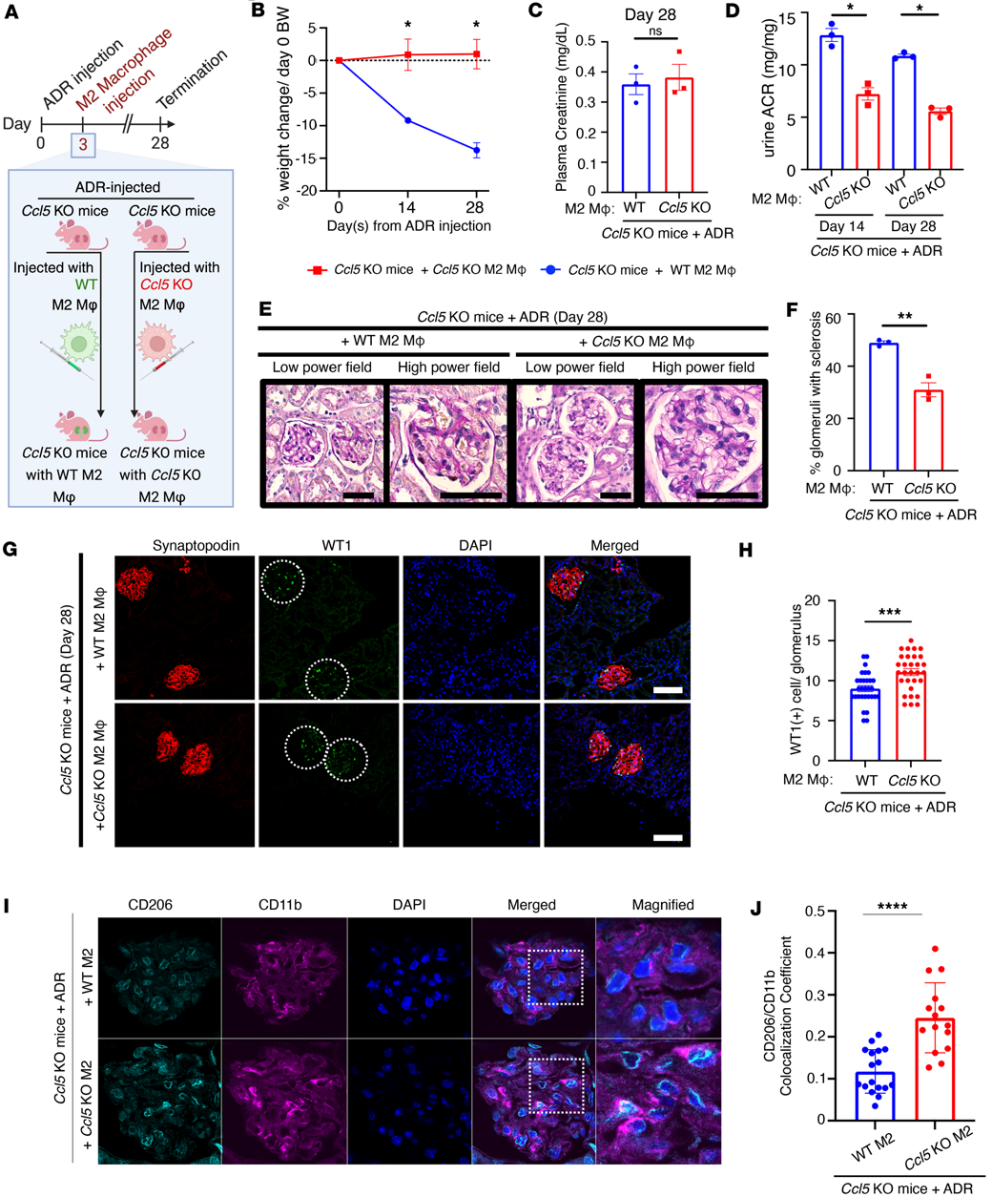
CCL5 in macrophages exacerbates ADR-induced nephropathy by limiting glomerular M2 macrophage accumulation. (**A**) Experimental diagram of the adoptive transfer of M2 macrophages in ADR-treated *Ccl5*-KO mice. ADR-treated *Ccl5*-KO mice were injected with WT M2 macrophages (*n* = 3) or with *Ccl5*-KO M2 macrophages (*n* = 3). (**B**) Percentage of body weight change from the initial body weight of ADR-induced nephropathy *Ccl5*-KO mice receiving WT M2 macrophages and *Ccl5*-KO M2 macrophages. (**C**) Plasma creatinine on day 28 after ADR injection. (**D**) Urine ACR on day 14 and 28 after ADR injection. (**E**) Representative PAS staining image of the kidney, sacrificed on day 28 after ADR injection. Scale bars: 50 μm. (**F**) Quantification of sclerotic glomeruli per total glomeruli. (**G**) Representative confocal microscopy analysis of WT-1 and synaptopodin immunofluorescent staining in ADR-induced nephropathy *Ccl5*-KO mice receiving WT M2 macrophages and *Ccl5*-KO M2 macrophages. Scale bars: 50 μm. (**H**) Quantification of the number of cells with positive WT-1 staining per glomerulus (10 glomeruli counted per mouse), analyzed by confocal microscopy of immunofluorescent staining. (**I**) Representative confocal microscopy analysis of CD11b and CD206 immunofluorescent staining in ADR-induced nephropathy *Ccl5*-KO mice receiving WT M2 macrophages or *Ccl5*-KO M2 macrophages. Original magnification, ×40. (**J**) Quantification of CD11b and CD 206 colocalization in ADR-induced nephropathy *Ccl5*-KO mice receiving WT M2 macrophages and *Ccl5*-KO M2 macrophages. All data are expressed as mean ± SEM. Unpaired 2-tailed *t* tests were performed to calculate the *P* values. *P < 0.05, **P < 0.01, ***P < 0.001, ****P < 0.0001. NS, not significant; Mφ, macrophages; urine ACR, urine albumin-to-creatinine ratio.

Macrophages play a crucial role in kidney repair, shifting between proinflammatory M1 and repair-promoting M2 phenotypes depending on the disease stage (26). Impaired M1-to-M2 transition can lead to persistent inflammation and tissue damage (27). To examine how CCL5 in macrophages influences glomerular injury, we induced ADR nephropathy in *Ccl5*-KO mice and injected them with M2 macrophages from either WT or *Ccl5*-KO mice (Figure 7A).

Following ADR injection, *Ccl5*-KO mice that received WT M2 macrophages experienced more severe weight loss compared with those injected with *Ccl5*-KO M2 macrophages (Figure 7B). Although there were no differences in plasma creatinine (Figure 7C), mice receiving WT M2 macrophages developed significantly more severe albuminuria (Figure 7D), a higher number of sclerotic glomeruli (Figure 7, E and F), and greater podocyte loss (Figure 7, G and H) than those injected with *Ccl5*-KO M2 macrophages. Masson’s trichrome staining further confirmed increased fibrotic lesions in glomeruli of mice receiving WT M2 macrophages compared with those receiving *Ccl5*-KO M2 macrophages (Supplemental Figure 9). These findings suggest that the presence of CCL5 in macrophages contributes to the exacerbation of ADR-induced glomerular injury.

To explore the underlying mechanisms, we assessed whether CCL5 in macrophages affects glomerular M2 macrophage presence. Immunofluorescent staining for CD11b and CD206 revealed significantly more CD11b^+^CD206^+^ cells in the glomeruli of mice injected with *Ccl5*-KO M2 macrophages than in those receiving WT M2 macrophages (Figure 7, I and J). These results indicate that CCL5 in BM-derived macrophages (BMDMs) actively limits M2 macrophage accumulation in the glomeruli, potentially skewing the immune environment toward a more proinflammatory phenotype that aggravates nephropathy.

### CCL5 skews macrophage polarization toward the M1 phenotype.

To investigate the mechanism by which CCL5 influences macrophage function in ADR nephropathy, we hypothesized that endogenous CCL5 promotes a proinflammatory macrophage profile by directing macrophage polarization toward the M1 phenotype, thereby reducing the reparative M2 population. To test this, we performed a series of in vitro polarization assays using BMDMs from WT and *Ccl5*-KO mice.

**Figure 8:**
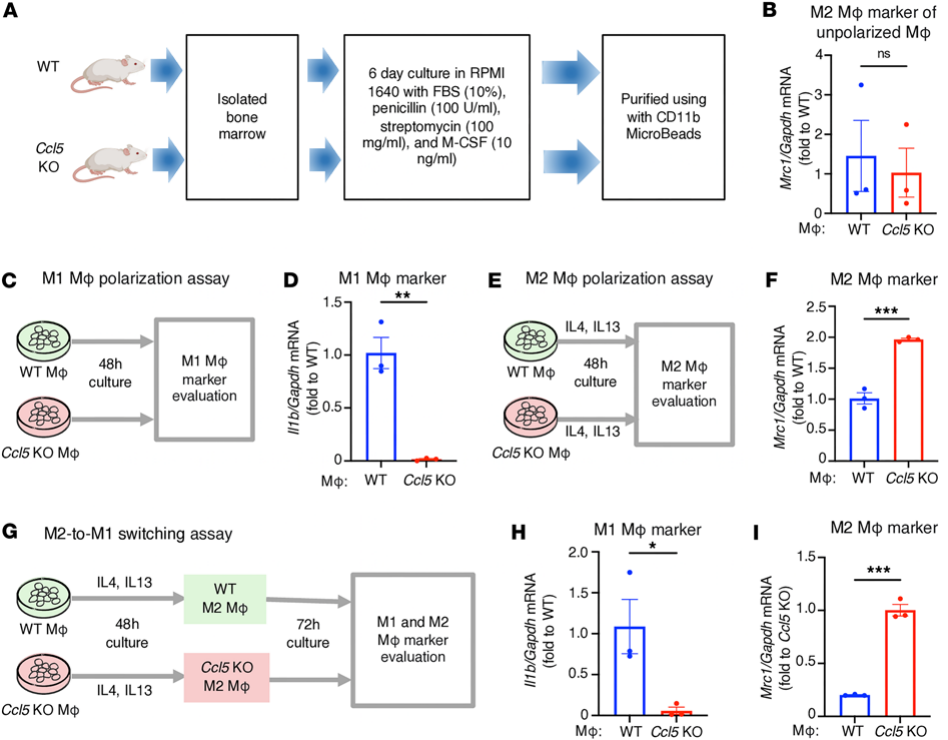
CCL5 skews macrophage polarization toward the M1 phenotype and inhibits M2 polarization. (**A**) Experimental diagram of macrophage isolation from BM cells of WT mice or *Ccl5*-KO mice. (**B**) Relative mRNA expression of M2 macrophage marker *Mrc1* in unpolarized macrophages from WT and *Ccl5*-KO mice. (**C**) Experimental diagram of the M1 macrophage polarization assay using macrophages from WT (*n* = 3) and *Ccl5*-KO mice (*n* = 3). (**D**) Relative mRNA expression of M1 macrophage marker *Il1b* in macrophages from WT and *Ccl5*-KO mice in M1 macrophage polarization assay. (**E**) Experimental diagram of the M2 macrophage polarization assay using macrophages from WT (*n* = 3) and *Ccl5*-KO mice (*n* = 3). (**F**) Relative mRNA expression of M2 macrophage marker *Mrc1* in macrophages from WT and *Ccl5*-KO mice in M2 macrophage polarization assay. (**G**) Experimental diagram of the M2-to-M1 shifting assay using macrophages from WT (*n* = 3) and *Ccl5-*KO mice (*n* = 3). (**H**) Relative mRNA expression of M1 macrophage marker *Il1b* in macrophages from WT and *Ccl5*-KO mice in M2-to-M1 shifting assay. (**I**) Relative mRNA expression of M2 macrophage marker in macrophages from WT and *Ccl5-*KO mice in M2-to-M1 shifting assay. The measured values were normalized to *Gapdh* and calculated using the ΔΔCt method. All data are expressed as mean ± SEM. Unpaired 2-tailed *t* tests were performed to calculate the *P* values. *P < 0.05, **P < 0.01, ***P < 0.001. NS, not significant; Mφ, macrophages; urine ACR, urine albumin-to-creatinine ratio.

BMDMs were cultured for 6 days, and macrophages were isolated using anti-CD11b magnetic beads with an AutoMACS separator (Figure 8A). Flow cytometry confirmed the purity of CD11b^+^ cells (Supplemental Figure 10). Prior to polarization, we measured the M2 macrophage marker *Mrc1* in unpolarized macrophages and found no significant differences between WT and *Ccl5*-KO macrophages (Figure 8B), indicating similar baseline M2 profiles.

We then conducted 3 polarization assays. First, to assess M1 polarization capacity, macrophages from WT and *Ccl5*-KO mice were treated with M1-polarizing stimuli (Figure 8C). WT macrophages showed significantly higher expression of M1 marker *Il1b* compared with *Ccl5*-KO macrophages (Figure 8D), suggesting that endogenous CCL5 promotes M1 polarization.

Second, to evaluate M2 polarization, WT and *Ccl5*-KO macrophages were exposed to M2-polarizing stimuli (IL-4 and IL-13) (Figure 8E). WT macrophages exhibited significantly lower expression of the M2 marker *Mrc1* than *Ccl5*-KO macrophages (Figure 8F); flow cytometry analysis of CD206 expression also showed similar results (Supplemental Figure 11). These results suggest that endogenous CCL5 suppresses M2 polarization.

Third, to examine the M2-to-M1 transition, we recultured previously polarized M2 macrophages from WT and *Ccl5*-KO mice without polarizing cytokines (Figure 8G). WT M2 macrophages demonstrated increased expression of the M1 marker *Il1b* and reduced expression of the M2 marker *Mrc1* compared with *Ccl5*-KO M2 macrophages (Figure 8, H and I), indicating a phenotypic shift from M2 to M1 in the presence of CCL5.

Together, these data demonstrate that endogenous CCL5 skews macrophage polarization toward the M1 phenotype by enhancing M1 polarization, inhibiting M2 polarization, and promoting M2-to-M1 transition. These mechanistic findings provide compelling evidence that CCL5 actively drives a proinflammatory macrophage phenotype, thereby limiting the reparative M2 response and amplifying immune-mediated glomerular injury in ADR-induced nephropathy.

## Discussion

CKD remains a major global health challenge, and understanding the mechanisms that drive its progression is essential for developing targeted therapies. In this study, we investigated the role of CCL5 in ADR-induced nephropathy, a widely used CKD model. Our findings reveal a paradoxical role of CCL5; it protects podocytes from apoptosis, yet promotes kidney injury through macrophage-driven inflammation. This cell-specific dichotomy highlights the complexity of chemokine signaling in glomerular disease and underscores the need for precision-targeted approaches.

Podocyte injury is a hallmark of many glomerular diseases, and its prevention is key to preserving kidney function. Our findings establish that CCL5 is upregulated in glomerular diseases in both human and mouse models. In patients with FSGS, IgAN, and LN, CCL5 showed prominent glomerular expression with a capillary pattern that colocalized with synaptopodin, a podocyte-specific marker. A similar expression pattern was observed in ADR-induced nephropathy in mice. CCL5 expression was minimal in normal glomeruli, as confirmed by immunofluorescent staining of histologically normal human kidney tissue, reinforcing that CCL5 upregulation is associated with injury rather than constitutive.

Our in vitro experiments revealed that CCL5 protects podocytes from ADR-induced apoptosis. Both exogenous CCL5 and endogenous overexpression significantly enhanced podocyte viability, whereas *Ccl5*-KO podocytes exhibited increased spontaneous and ADR-induced apoptosis. These results indicate that podocytes not only express CCL5 but also respond to it in a survival-promoting manner. To exclude off-target effects of CRISPR/Cas9 editing, we performed in silico analysis using Cas-OFFinder, which revealed no predicted off-targets in genes related to kidney function. Sanger sequencing confirmed a precise 37–base pair deletion at the intended target site without unexpected nearby mutations. Together with our rescue experiments using exogenous CCL5, these findings confirm that the observed phenotypes result from specific *Ccl5* deletion rather than off-target events. While transient knockdown approaches (e.g., siRNA or CRISPRi) may further clarify the acute roles of CCL5, our study emphasizes the long-term consequences of stable deletion. Further studies should explore alternative gene-silencing methods and assess potential compensatory pathways that may influence CCL5’s effects.

Our analysis of CCL5 receptor expression showed that GPR75 was the only one expressed in cultured podocytes. Prior studies demonstrated that CCL5/GPR75 signaling activates the PI3K/Akt pathway to promote neuroprotection (28, 29). We propose that CCL5 has a similar mechanism in podocytes, promoting survival via PI3K/Akt signaling and Bcl-2 phosphorylation. Although we did not directly test this pathway, our findings suggest its relevance. Future work should explore whether GPR75 activation drives PI3K/Akt signaling and confirm the role of this axis in CCL5-mediated cytoprotection. It remains unclear whether CCL5 acts through autocrine or paracrine signaling, or whether other mediators such as 20-hydroxyeicosatetraenoic acid (20-HETE) are involved. Although we did not use GPR75 inhibitors, shRNA, or 20-HETE antagonists, we acknowledge this as a limitation and suggest further studies to dissect these mechanisms.

CCL5’s context-dependent effects have been reported across various systems. While it promotes inflammation in atherosclerosis, liver fibrosis, and inflammatory airway disorders (30–32), it also supports astrocyte survival and regulates insulin secretion in the hypothalamus (33, 34). Although CCL5 protected in vitro, mice lacking CCL5 experienced less severe ADR-induced nephropathy. This suggests that, within the kidney as a whole, the harmful proinflammatory effects of CCL5 outweigh its protective function in podocytes.

ADR-induced nephropathy follows a biphasic disease course, with early podocyte injury and proteinuria followed by later development of glomerulosclerosis and fibrosis (35). CCL5 may protect podocytes during early injury, as shown in our in vitro data, but contribute to sustained inflammation and fibrosis at later stages. This temporal shift may explain the paradoxical roles of CCL5 depending on disease stage and cellular context. Further time-course studies are needed to elucidate these transitions.

Given a central role of macrophages in ADR-induced nephropathy, we hypothesized that CCL5 promotes injury by modulating macrophage polarization. Macrophages are highly plastic cells, capable of shifting between proinflammatory M1 and reparative M2 phenotypes. We found that CCL5 deficiency increased glomerular M2 macrophages and reduced kidney injury. These findings support the role of M2 macrophages in promoting resolution and repair (36, 37). Moreover, earlier research demonstrated that impaired M1-to-M2 transition leads to persistent renal inflammation and fibrosis (38). Even a small number of M1 macrophages can worsen kidney injury in ADR nephropathy models (39). Our results also align with prior research showing that M2-to-M1 transition worsens disease progression (40). These findings indicate that CCL5 drives macrophage polarization toward a proinflammatory phenotype.

BMT experiments clarified CCL5’s source-specific effects. Mice receiving *Ccl5*-deficient BM exhibited reduced albuminuria, less glomerulosclerosis, and higher WT-1^+^ podocyte counts, regardless of host genotype, suggesting that BM-derived CCL5 drives injury. In contrast, *Ccl5*-KO host mice exhibited greater injury, indicating a protective role for kidney-resident CCL5. The most severe phenotype occurred in mice with CCL5 restricted to BM-derived cells, while the mildest phenotype occurred in those with CCL5 only in kidney-resident cells.

To minimize sex-related bias, both male and female mice were used in the ADR-injected WT and *Ccl5*-KO groups. However, due to technical constraints, only female mice were used as hosts and male mice as donors in the BMT experiments, while all mice in the M2 macrophage transfer experiments were male. Although analyses were not stratified by sex, future studies should evaluate potential sex-based differences in CCL5’s effects on glomerular injury and macrophage function.

To directly evaluate the contribution of macrophage-derived CCL5, we transferred M2 macrophages from WT or *Ccl5*-KO mice into ADR-injected *Ccl5*-KO recipients. Mice receiving WT M2 macrophages had worse clinical and histological outcomes and fewer glomerular M2 macrophages. This suggests that endogenous CCL5 in macrophages destabilizes the M2 phenotype. Our in vitro assays confirmed that CCL5 promotes M1 polarization, inhibits M2 polarization, and drives M2-to-M1 conversion. Importantly, M2 marker expression in unpolarized WT and *Ccl5*-KO macrophages was similar, indicating that CCL5 influences macrophage fate dynamically in response to environmental cues. Prior studies support this mechanism. Li et al. showed that pharmacological inhibition of CCL5 reduced M1 markers and increased M2 polarization in a model of drug-induced liver injury, reinforcing that CCL5 promotes proinflammatory macrophage phenotypes (41). Another study in drug-induced liver injury also demonstrated that CCL5 drives M1 polarization via the MAPK and NF-κB pathways, acting through CCR1 and CCR5 receptors (40). Given that these pathways are central to M1 macrophage activation (40–42), we propose that in BMDMs, CCL5 activates MAPK and NF-κB signaling via CCR1 and CCR5, leading to sustained M1 polarization and kidney damage. These findings suggest that blocking CCL5 signaling through CCR1 or CCR5 could represent a targeted approach to limit inflammation in ADR nephropathy while avoiding disruption of its protective role in podocytes.

While CD11b and CD206 costaining effectively identified glomerular M2 macrophages, CD11b alone is not macrophage specific, and in vivo identification of M1 macrophages remains limited by the absence of reliable immunofluorescence markers. Broader macrophage profiling using markers such as CD68 or F4/80 could enhance the assessment of total macrophage burden. Although we did not perform additional immunostaining due to the scope of this study, we acknowledge this as a limitation to be addressed in future investigations.

Although our study emphasizes the beneficial role of M2 macrophages in the ADR model, other studies have reported context-dependent detrimental effects. For example, CASK-mediated M2 activation was linked to fibrosis in FSGS (43). In contrast, our findings, along with prior studies, demonstrate that early M2 polarization reduces proteinuria and glomerulosclerosis (44). Further research is needed to explore whether prolonged M2 activation eventually leads to maladaptive remodeling in later stages.

This study used a single CKD model — ADR-induced nephropathy, which reflects FSGS but may not represent the pathophysiology of other CKD subtypes such as diabetic nephropathy or LN. However, CCL5 was upregulated in multiple human glomerular diseases, including FSGS, IgAN, and LN, suggesting broader clinical relevance. The discrepancy between injury models may reflect differences in the nature, duration, and immune context of injury. In acute models of injury, CCL5 has been reported to facilitate early repair (45, 46). In contrast, in other chronic disease, sustained CCL5 expression contributes to maladaptive inflammation by promoting macrophage polarization toward a proinflammatory phenotype (47). This highlights the importance of further studies in diverse CKD models to clarify the generalizability of our findings.

Regarding the quantification of podocyte loss, we used WT-1 staining per glomerular cross section without applying the correction factor proposed by Venkatareddy et al., which adjusts for section thickness and nuclear diameter (48). Although our method is widely used (49, 50) and applied consistently across groups, more accurate estimation of podocyte density using this correction would strengthen future studies.

In summary, this study highlights the dual and context-dependent role of CCL5 in ADR-induced nephropathy. CCL5 protects podocytes from apoptosis yet promotes glomerular injury through macrophage polarization. These opposing effects emphasize the need for targeted interventions. Rather than global CCL5 inhibition, selective CCR1/CCR5 antagonism in macrophages may preserve the podocyte-protective effects of CCL5 while mitigating its proinflammatory activity, offering a more refined therapeutic strategy for glomerular disease.

## Methods

### Sex as a biological variable.

Our study examined both male and female mice. In the ADR-injected WT and *Ccl5*-KO groups, similar proportions of male and female animals were used to minimize sex-related bias, and similar findings were observed across sexes. In BMT experiments, female mice were used as hosts and male mice as donors due to technical constraints related to engraftment efficiency. In adoptive transfer experiments involving M2 macrophages, only male mice were used for consistency. While we did not stratify our analyses by sex, the main findings were consistent across groups and are expected to be relevant for both sexes.

### ADR-induced nephropathy mouse model.

Eight-week-old male and female BALB/c *Ccl5*-KO (*Ccl5*^–/–^) mice and WT (*Ccl5*^+/+^) littermates were injected with ADR (doxorubicin hydrochloride; Wako) (10 mg/kg, i.v.), as previously described (16). Mice were sacrificed on day 28 after injection of ADR, and the kidneys were harvested.

### Generation of BALB/c Ccl5-KO mice.

*Ccl5*-KO mice were purchased from The Jackson Laboratory (B6.129P2-*Ccl5^tm1Hso^*/J), and BALB/c mice were purchased from CLEA Co. The C57BL6 *Ccl5*^–/–^ male mouse was mated with a female BALB/c mouse to generate heterozygous N1. The male heterozygous N1 mice were backcrossed to the BALB/c background for 8 generations. Then, backcrossed *Ccl5*^+/–^ were crossed with *Ccl5*^+/–^ to generate *Ccl5*^–/–^ and *Ccl5*^+/+^. The *Ccl5*^+/+^ mice served as WT control. These backcrossed mice were then used for experiments and are described here as *Ccl5*-KO and WT mice.

### Generation of BMT mice.

BMT mice were generated by transplanting BM cells (2 × 10^7^ cells) from male mice into female host mice 6 hours after the host received lethal irradiation. The host mice received a total radiation dose of 9.5 Gy in two 4.75 Gy fractions separated by an interval of approximately 12 hours. Eight weeks after reconstitution, donor BM engraftment was validated using qPCR to evaluate the *Zfy1* gene, the specific gene of the Y chromosome, as previously described (51).

### ADR-induced nephropathy murine model in BMT mice.

ADR (10 mg/kg, i.v.) was injected into validated BMT mice 8 weeks after BMT to establish ADR-induced nephropathy. The mice were then sacrificed on day 28 after ADR injection for analysis.

### Macrophage isolation and polarization.

Primary murine macrophage cultures were obtained from the BM of male *Ccl5*-KO and WT mice, as previously described (52). BMDMs were generated by culturing primary BM cells isolated from *Ccl5*-KO and WT mice in RPMI 1640 medium, containing fetal bovine serum (10%), penicillin (100 U/mL), streptomycin (100 mg/mL), and M-CSF (10 ng/mL) for 6 days, and then purifying with CD11b MicroBeads (Miltenyl Biotec). To analyze the purity of the obtained BMDMs, cells were stained with FITC anti–mouse F4/80 (BioLegend), PE anti–mouse MHC-II (BioLegend), anti-CD206 (Novus Biologicals), and BV421 anti–mouse CD163 (BioLegend) (see Supplemental Table 3). The purity was estimated using FACS analysis by evaluating the percentage of positive cells using the FlowJo software (BD Life Sciences) program. For the M1 polarization assay, the purified BMDMs were cultured with medium for 48 hours. For the M2 polarization assay, the purified BMDMs were cultured with medium plus IL-4 and IL-13 (10 ng/mL each; R&D Systems) for 48 hours. For M2-to-M1 transition assay, the polarized M2 macrophages were washed and cultured with medium without IL-4 and IL-13 for 72 hours. The phenotypes of macrophages were examined by qPCR by evaluating the relative mRNA expression of *Il1b* as the M1 macrophage marker or *Mrc1* as the M2 macrophage marker. The polarized M2 macrophages were also analyzed using flow cytometry by evaluating CD206 and CD163.

### Adoptive transfer of macrophages to ADR-induced nephropathy mice.

*Ccl5*-KO mice were injected with ADR (10 mg/kg, i.v.). Three days after ADR injection, polarized M2 macrophages (1 × 10^6^ cells) from *Ccl5*-KO or WT mice were injected into ADR-injected *Ccl5*-KO mice. Mice were sacrificed 4 weeks after injection of ADR. The mice were all kept under specific pathogen–free conditions.

### Immunofluorescence analysis.

Immunofluorescent staining and analysis of mouse kidneys were performed as previously described (53). Glomeruli with sclerosis was measured by quantifying the total number of glomeruli per section and determined the percentage of glomeruli exhibiting segmental or global sclerosis based on histological staining. Analysis was performed in a blinded manner to reduce bias. The antibodies used in this investigation are listed in Supplemental Table 3. Immunofluorescence images of frozen mouse kidney sections were obtained using an LSM 780 microscope (Zeiss).

### Human kidney biopsy samples.

Human kidney biopsy samples were obtained from Chiba University Hospital.

### Measurement of urine albumin, urine creatinine, and serum creatinine.

Urinary albumin was measured using an LBIS mouse albumin ELISA kit (FUJIFILM Wako) and urine and plasma creatinine were measured using LabAssay Creatinine (Wako).

### Histological analysis.

The kidneys were removed, cut longitudinally in half, and fixed with 4% paraformaldehyde (PFA) in PBS at 4°C overnight. After dehydration, the kidneys were embedded in paraffin. Paraffin blocks were sectioned to 2.5 μm thicknesses and stained with periodic acid–Schiff (PAS) for histological evaluation.

### Western blot.

Podocyte lysates were prepared in 0.5% CHAPS buffer and supplemented with protease inhibitor (Complete Mini; Roche Applied Science) and phosphatase inhibitor (PhosSTOP; Roche Applied Science). The proteins were eluted with 4× Laemmli buffer/2-mercaptoethanol. Western blotting and SDS-PAGE were conducted as previously described (54). Supplemental Table 3 contains a list of antibodies used in this study.

### RNA extraction and qPCR.

RNA extraction and qPCR were performed as previously described (16). All measured values were normalized to *Gapdh* and calculated using the ΔΔCt method. The primers in this study are described in Supplemental Table 4.

### Analysis of secreted CCL5 concentration by ELISA.

Secreted CCL5 concentration was measured using a mouse/rat CCL5/RANTES quantikine (R&D Systems) ELISA kit according to the manufacturer’s protocol.

### Cell culture.

Conditionally immortalized murine podocyte culture was performed as previously explained (55). To induce CCL5 expression, podocytes were treated with 0.15 μg/mL ADR or a combination of 0.15 μg/mL ADR and 50 μg/mL αNotch2 (provided in-house) for 6 hours. To induce CCL5 secretion, podocytes were treated with 0.15 μg/mL ADR or a combination of 0.15 μg/mL ADR and 50 μg/mL αNotch2 for 12 hours. To induce apoptosis, podocytes were treated with 0.15 μg/mL ADR. Mouse recombinant CCL5 protein (BioLegend) was administered at concentrations of 5 nM, 25 nM, and 50 nM to investigate the effect of CCL5 on podocyte apoptosis.

### Apoptosis assay.

Apoptosis assays were performed on WT and genome-edited podocytes treated with 0.15 μg/mL ADR or a combination of 0.15 μg/mL ADR plus mouse recombinant CCL5 protein (BioLegend) that was administered at concentrations of 5 nM, 25 nM, or 50 nM. Cells were collected 12 hours after ADR treatment. Cells were washed, suspended, and stained with APC–Annexin V and 7-AAD at a concentration recommended by the manufacturer. At least 10,000 cells per sample were analyzed in a FACSAria III (BD) and then analyzed using FCS Express 7 (De Novo Software). Apoptotic cells were identified as Annexin V^+^ cells and viable cells as Annexin V^–^7-AAD^–^ cells.

### Plasmid constructs.

A full-length cDNA clone of mouse *Ccl5* was obtained from Sino Biological and cloned in-frame into pFLAG-CMV-5a vectors (Sigma-Aldrich). Empty vectors were used as negative controls. All constructs were verified by Sanger sequencing.

### Establishment of a CCL5-OE podocyte cell line.

To establish CCL5-OE cultured podocytes, a piggyBac transposon system was applied (56). The GFP sequence was removed from the pPV-EF1α-EiP-A piggyBac vector (provided in-house), and FLAG-tagged *Ccl5* cDNA was cloned into the vector (pPB-EF1α-CCL5-FLAG-EiP-A). On the other hand, a control podocyte, pPV-EF1α-Control-EiP-A, was constructed using inverse PCR. These piggyBac vectors and pHL-EF1a-hcPBase (piggyBac transposed expression vector) were cotransfected into undifferentiated cultured podocytes via lipofection. Forty-eight hours after transfection, puromycin selection (1.25 μg/mL) was applied. Four days after selection, the remaining puromycin-resistant colonies were dissociated into single cells. After the expansion of each single cell clone, CCL5 expression was confirmed by Western blotting and ELISA.

### Establishment of a Ccl5-KO podocyte cell line.

CRISPR/Cas9 technology was used to generate *Ccl5*-KO podocyte cell lines. Based on previous studies, we designed 3 guide RNAs for exon 1 of *Mus* *musculus* *Ccl5* (GAGACCACTTGGATCCGGCAGAGGGCGGCTGCAGTGGTTTTAGAGCTAGAAATAGCA, GAGACCACTTGGATCCGGCAGGTGCGGGGGTGCAGAGTTTTAGAGCTAGAAATAGC, and GAGACCACTTGGATCCGACATGGTGAGGCAGGTGCGGTTTTAGAGCTAGAAATAGCA) using the web-based tool CRISPRdirect (https://crispr.dbcls.jp) (57). Bioinformatic analysis using Cas-OFFinder was also performed to analyze the potential off-target sites of this sgRNA. The guide RNA sequence was cloned into a target vector (pHL-H1-ccdB-mEF1a-RiH; Addgene plasmid 60601), following the recommendations of the vector developers (58). The subcloned and Cas9 expression vectors (pHL-EF1a-SphcCas9-iP-A; Addgene plasmid 60599) were transfected into mouse cultured podocytes with a lipofection method. The original target vector without the guide RNA sequence and the Cas9 expression vector were transfected for control podocytes. Two days after transfection, hygromycin (125 μg/mL) and puromycin selection (0.625 μg/mL) was applied for 72 hours. Based on the results of the T7 endonuclease I assay (EnGen Mutation Detection Kit, New England Biolabs), the remaining hygromycin- and puromycin-resistant colonies were dissociated into single cells. After the expansion of each single-cell clone, KO of *Ccl5* was confirmed using Sanger sequencing, Western blotting, and ELISA. Potential off-target mutations were also examined by Sanger sequencing.

### Statistics.

Data are presented as mean ± SEM. The unpaired *t* test was used to compare 2 data sets, while 1-way analysis of variance (ANOVA) was used to compare multiple data groups unless indicated differently in the figure. Significant results were considered when *P* was less than 0.05.

### Study approval.

All protocols for animal experiments were approved by Chiba University’s Review Board for Animal Care and performed according to the Chiba University’s Guidelines for Animal Experiments (Chiba University’s Review Board for Animal Care 5-36,37). The study using human kidney biopsy samples was conducted with written informed consent and was approved by the ethics committee on human research of Chiba University Hospital (Chiba University Hospital no. 1178).

### Data availability.

The experimental data are available from the corresponding author upon request. Values for all data points in graphs are reported in the Supporting Data Values file.

## Author contributions

INK and KA conceived the hypothesis and conducted the experiments. INK and KA wrote the manuscript. INK, IO, and KY conducted the experiments and analyzing data. MAE, H Yamada, SM, RKN, NT, MYK, and MA collaborated on the experiments and provided technical advice. INK, MAE, and KA revised the manuscript. H Yagita provided the Notch2 agonist antibody. AH provided the plasmid vectors. KA supervised the experimental design and interpreted the results. INK, IO, and KY are co–first authors. The order of authorship among these co–first authors was determined based on the relative extent of their contributions to hypothesis development, experimental work, data analysis, and manuscript preparation.

## Funding support

Ministry of Education, Culture, Sports, Science and Technology of Japan Grant-in-Aid for Scientific Research (grants 17K19653, 18H02823 and 23H02923 to KA and 21K16158 to NT).

- Indonesian Endowment Fund (grant 201710220411855 to INK).

## Supplementary Material

Supplemental data

Unedited blot and gel images

Supporting data values

## Version 1 09/23/2025

In-Press Preview

## Version 2 11/10/2025

Electronic publication
