# Supplementary material for: CCL5 paradoxically regulates glomerular injury by skewing macrophage polarization
Source: JCI Insight. 2025 Sep 23;10(21):e173742. doi: 10.1172/jci.insight.173742 (PMC12643500; doi:10.1172/jci.insight.173742)
Supplement: Supplemental data [file jciinsight-10-173742-s107.pdf]

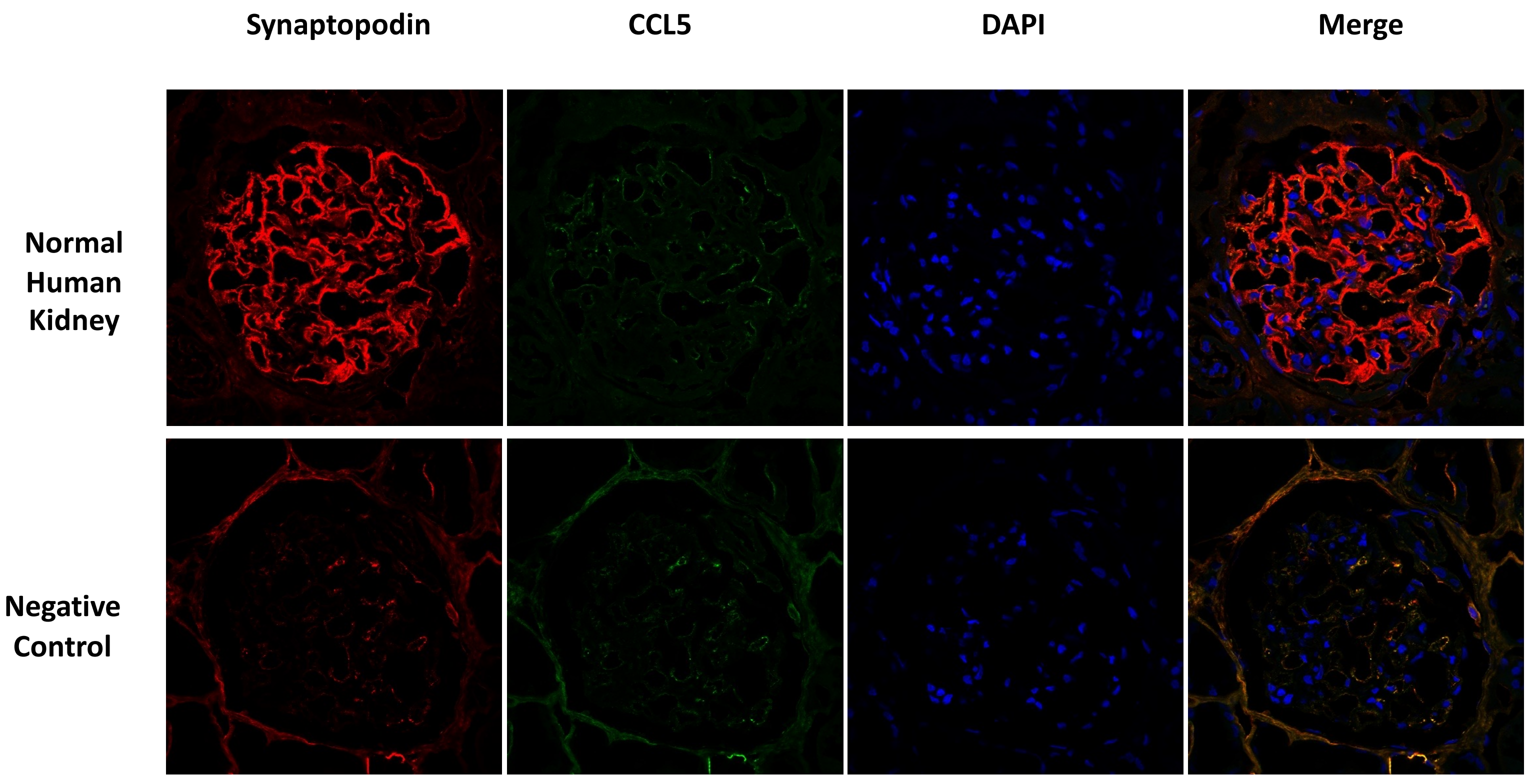

Figure S1. Immunofluorescence staining of CCL5 in normal human kidney samples. Representative images of normal human kidney tissue stained for CCL5 (green) and the podocyte marker synaptopodin (red). CCL5 expression was minimal to undetectable in normal glomeruli, suggesting that CCL5 is expressed at low levels under physiological conditions but becomes upregulated in response to glomerular injury. Nuclei were counterstained with DAPI (blue). These findings serve as a baseline reference for CCL5 expression in healthy kidneys, supporting the disease-specific upregulation observed in glomerular disease and ADR-induced nephropathy models.

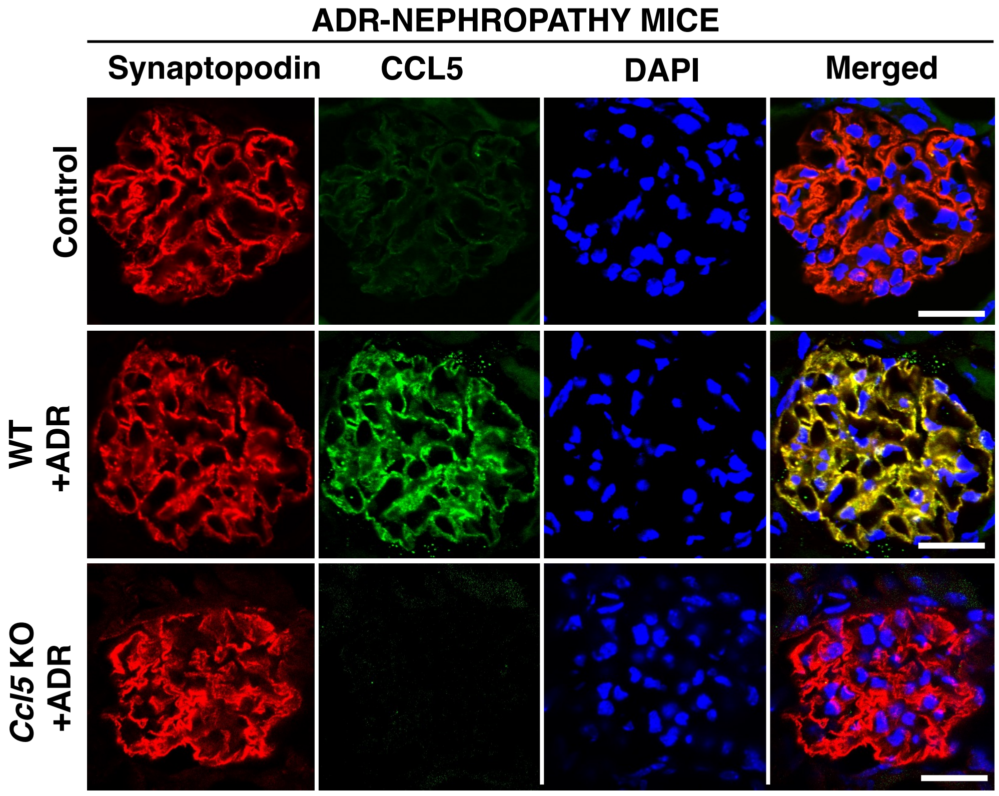

Figure S2. Immunofluorescence staining of CCL5 in the kidney of ADR-injected *Ccl5* KO mice. Representative images show CCL5 immunofluorescence staining in kidney sections from ADR-injected WT mice and ADR-injected *Ccl5* KO mice. The absence of detectable CCL5 staining in ADR-injected *Ccl5* KO mice confirms the knockout of *Ccl5* in this model and validates the specificity of the CCL5 antibody.

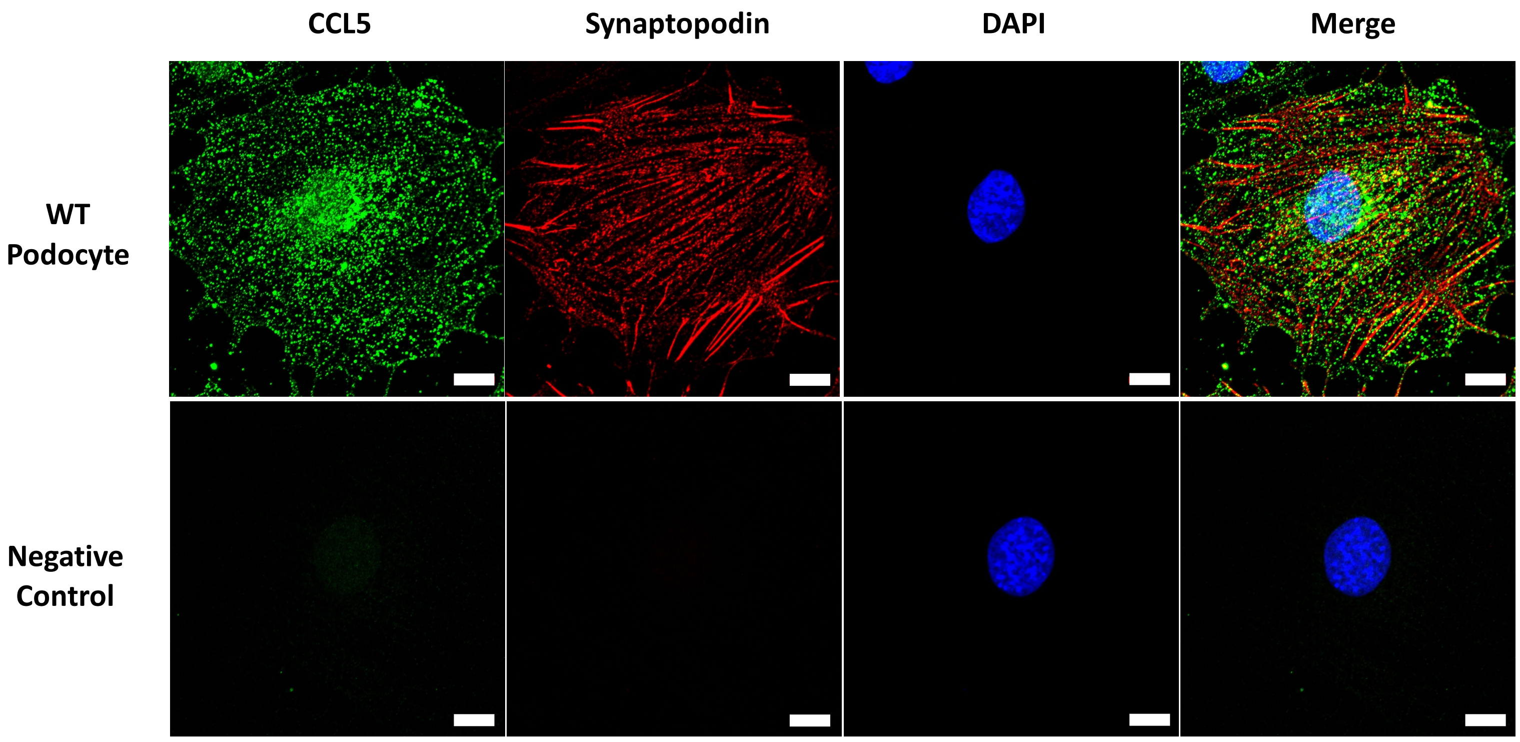

Figure S3. Immunofluorescence staining of CCL5 in wild-type cultured podocytes. Representative immunofluorescence images of wild-type cultured podocytes stained for CCL5 (green) and the podocyte marker synaptopodin (red). CCL5 colocalizes with synaptopodin, confirming its intracellular expression in podocytes under baseline conditions. Nuclei were counterstained with DAPI (blue). These findings support that podocytes are a source of CCL5 even in the absence of injury, complementing the mRNA and protein secretion analyses presented in the main figures. Scale bar: 10µm.

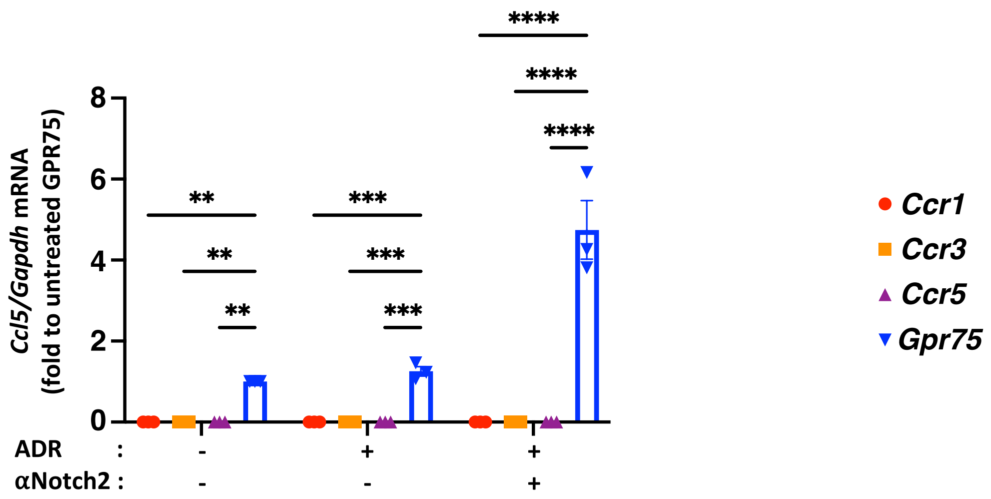

Figure S4. mRNA Expression of *Ccl5*'s receptors in cultured podocytes. Conditionally immortalized murine cultured podocytes were received no treatments or stimulated with 0.15 mg/ml of ADR or combination of 0.15 mg/ml of ADR and 50mg/ml of αNotch2. Relative mRNA expression of *Ccl5* receptors in cultured podocytes. Measured values were normalized to *Gapdh* and calculated by the △△CT method. **P<0.01; ***P<0.001; ***P<0.0001.

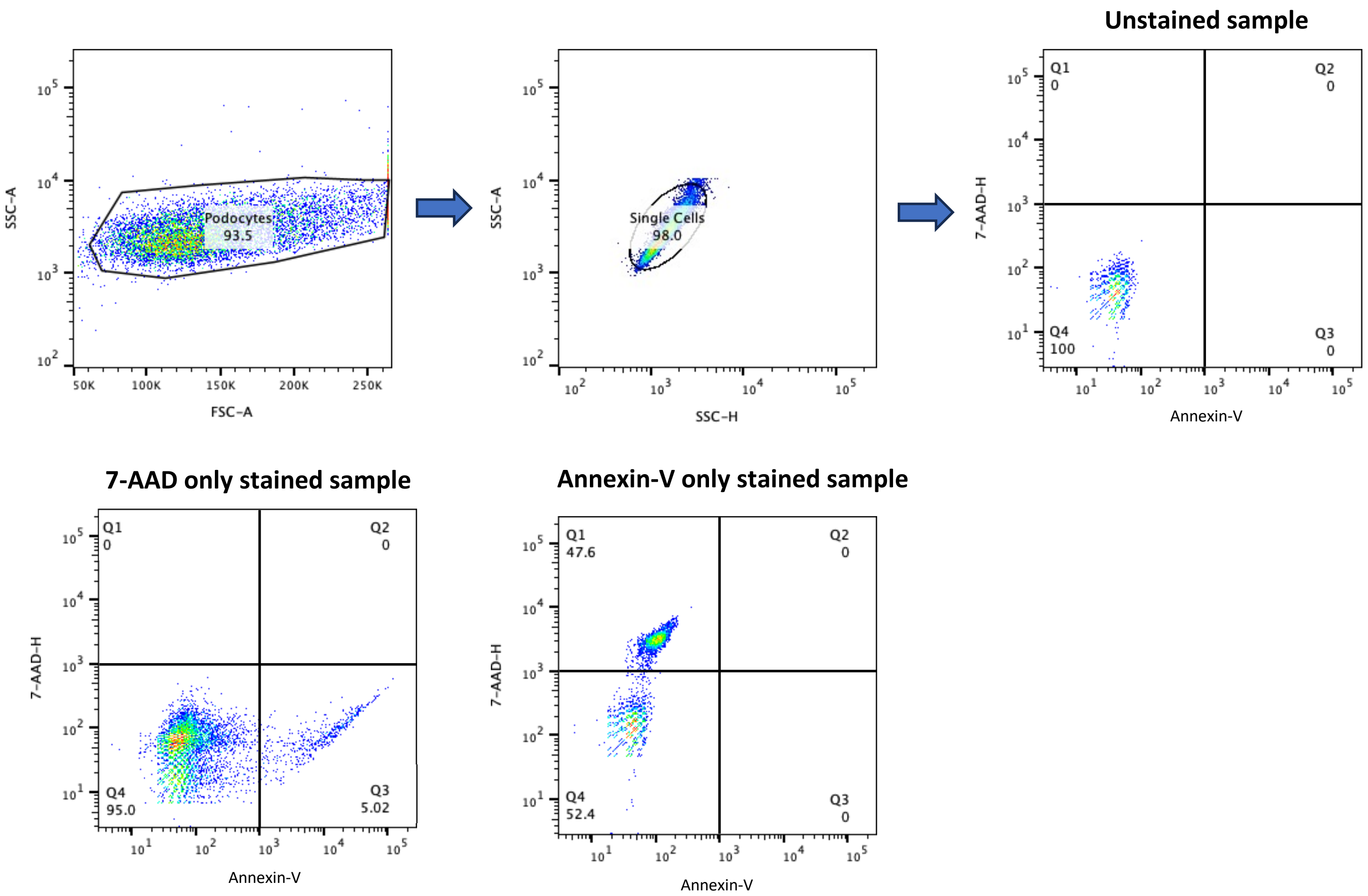

Figure S5. Full gating strategy for Annexin V/7-AAD flow cytometry. Flow cytometry plots showing FSC vs SSC gating, unstained control, single-stained controls (Annexin V only, 7-AAD onlywith quadrant gating. A minimum of 10,000 events were collected per sample.

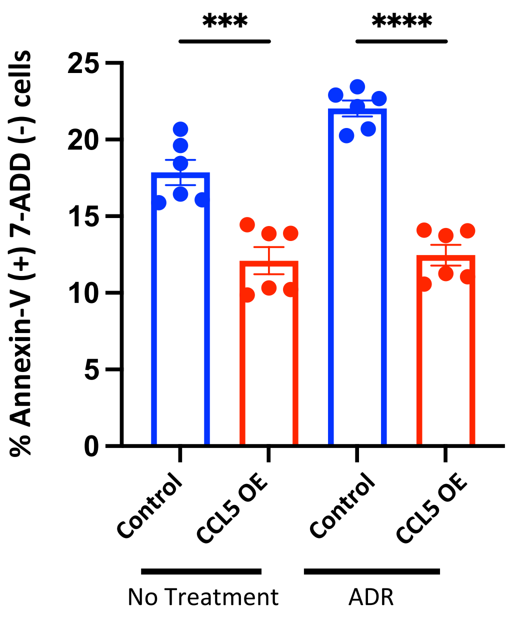

Figure S6. Analysis of early apoptotic events (Annexin V$^+$/7-AAD$^-$) in control and CCL5-overexpressing (CCL5 OE) podocytes. Flow cytometry data show the percentage of early apoptotic cells in untreated and ADR-treated conditions. Data are presented as mean ± SEM. ***P < 0.001; ****P < 0.0001 by unpaired t-test..

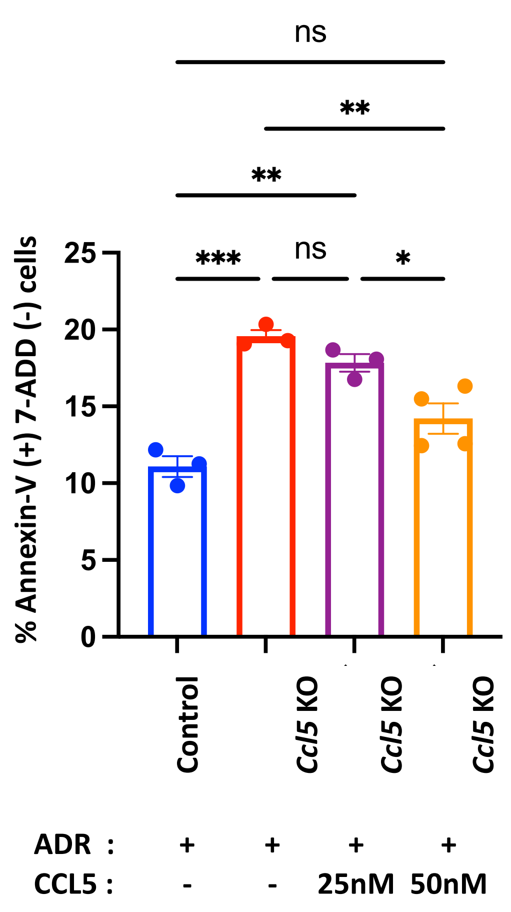

Figure S7. Analysis of early apoptotic events (Annexin V^{+}/7-AAD^{-}) in *Ccl5* KO podocytes treated with ADR, with or without exogenous CCL5. Flow cytometry data show the percentage of early apoptotic cells in *Ccl5* KO podocytes treated with ADR, with or without exogenous CCL5. Data are presented as mean ± SEM. *P < 0.05; **P < 0.01 by one-way ANOVA with Tukey's post hoc test.

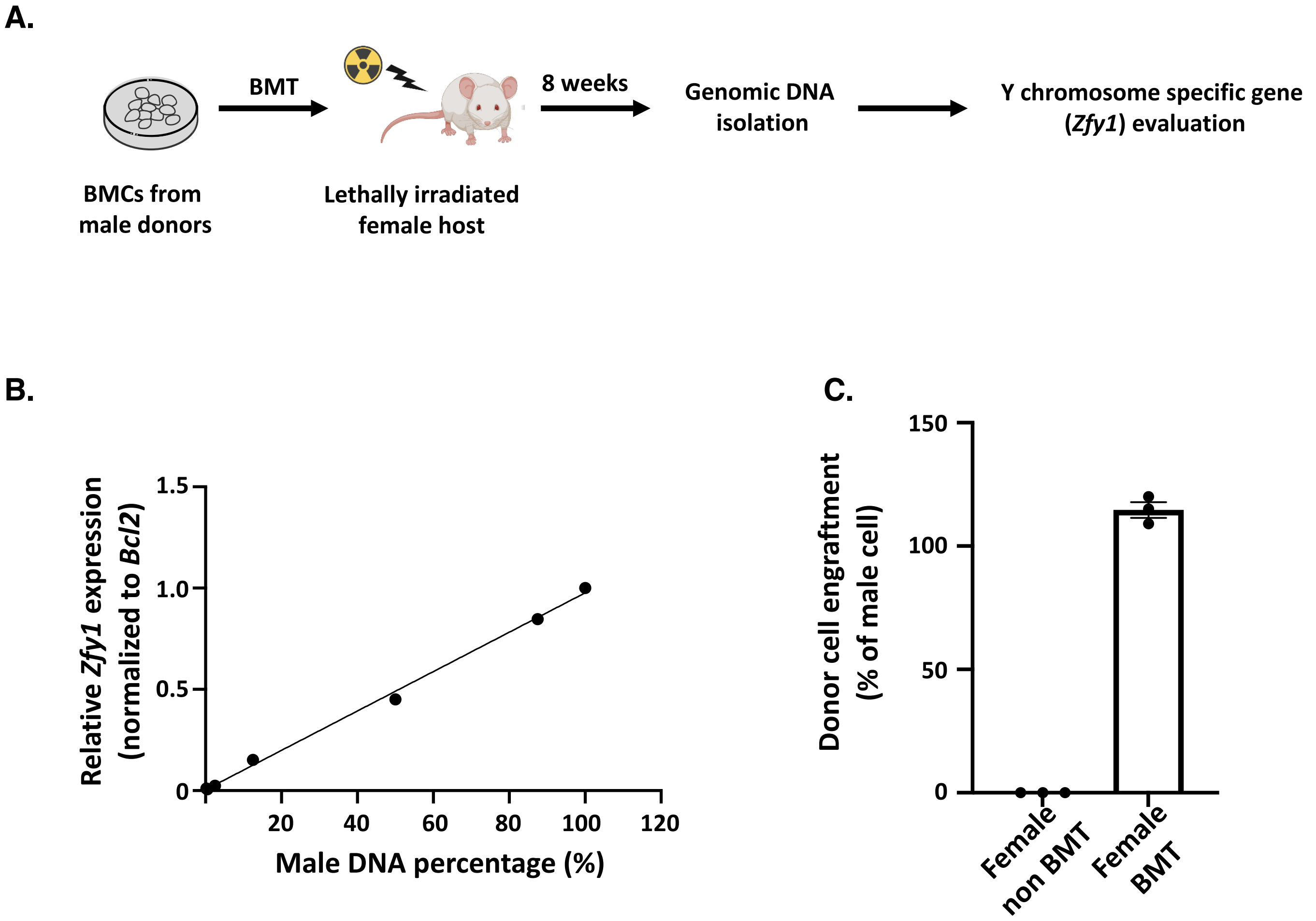

Figure S8. Validation of bone marrow cells engraftment in the BMT mice (A) Schematic diagram of bone marrow cells engraftment by real time PCR. (B) Representative standard curve for male DNA percentage. (C) Representative results of donor cell engraftment validation.

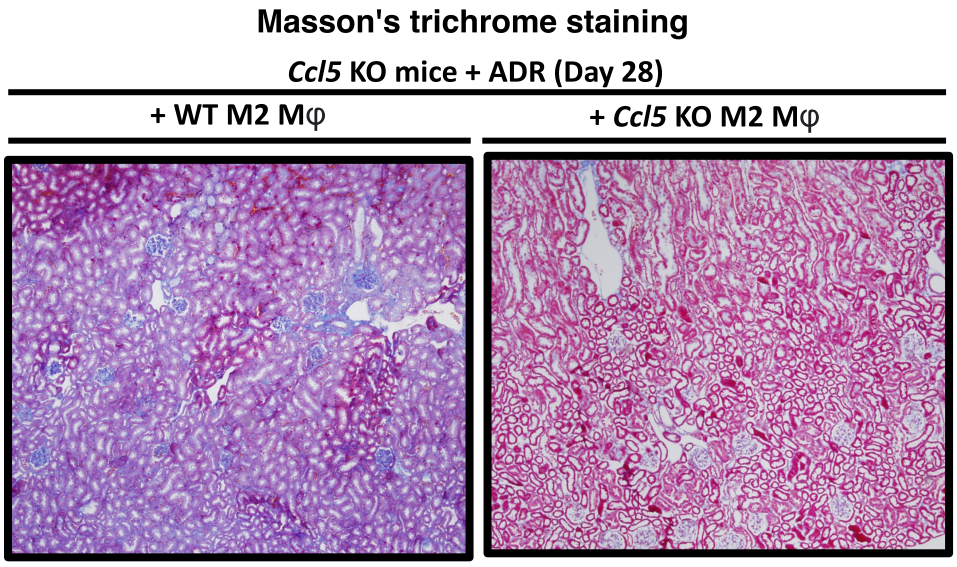

Supplementary Figure S9. Representative images of Masson trichrome staining of kidney sections from *Ccl5* KO mice treated with Adriamycin (ADR) and injected with either wild-type (WT) M2 macrophages or *Ccl5* KO M2 macrophages. Kidney tissues were harvested and analyzed 28 days post-ADR injection. WT M2-injected mice showed more severe glomerulosclerosis, indicated by increased blue collagen deposition, compared to mice that received *Ccl5* KO M2 macrophages.

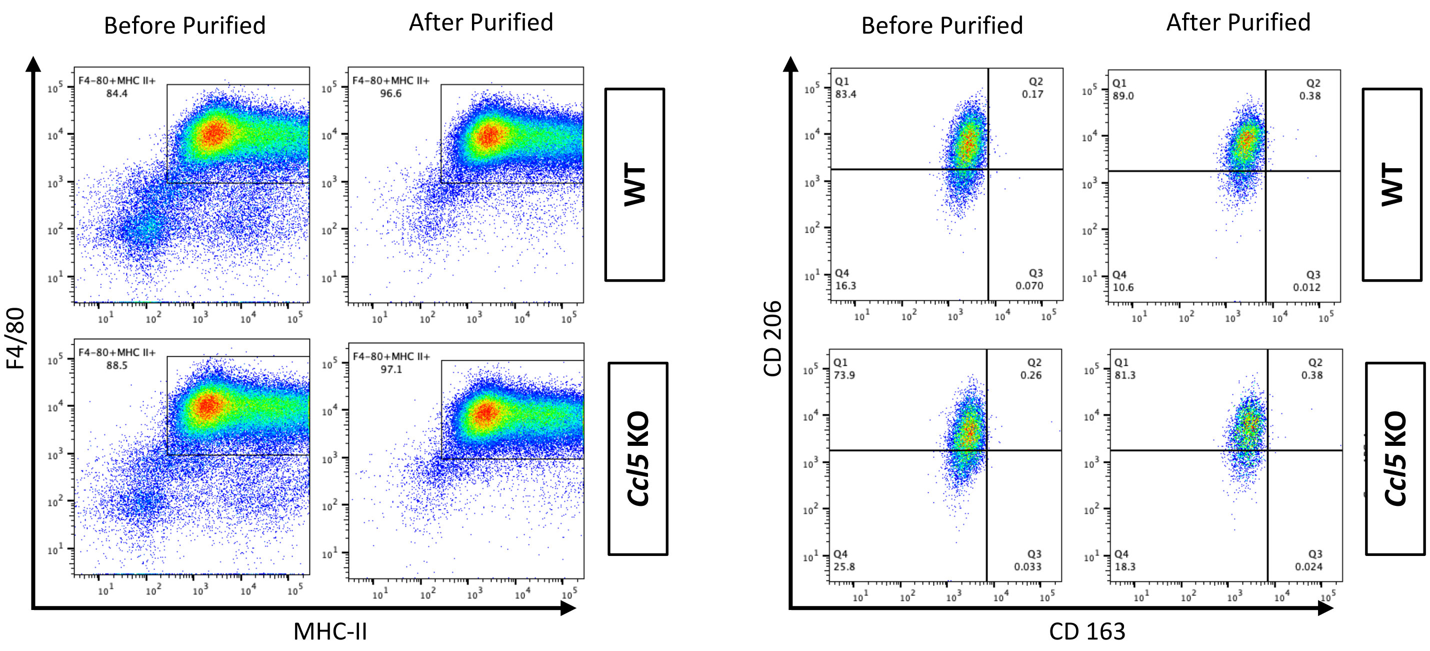

Figure S10. Purity analysis of isolated cultured macrophage. Representative results of purity analysis of unpolarized macrophages after purification using AutoMACS separator.

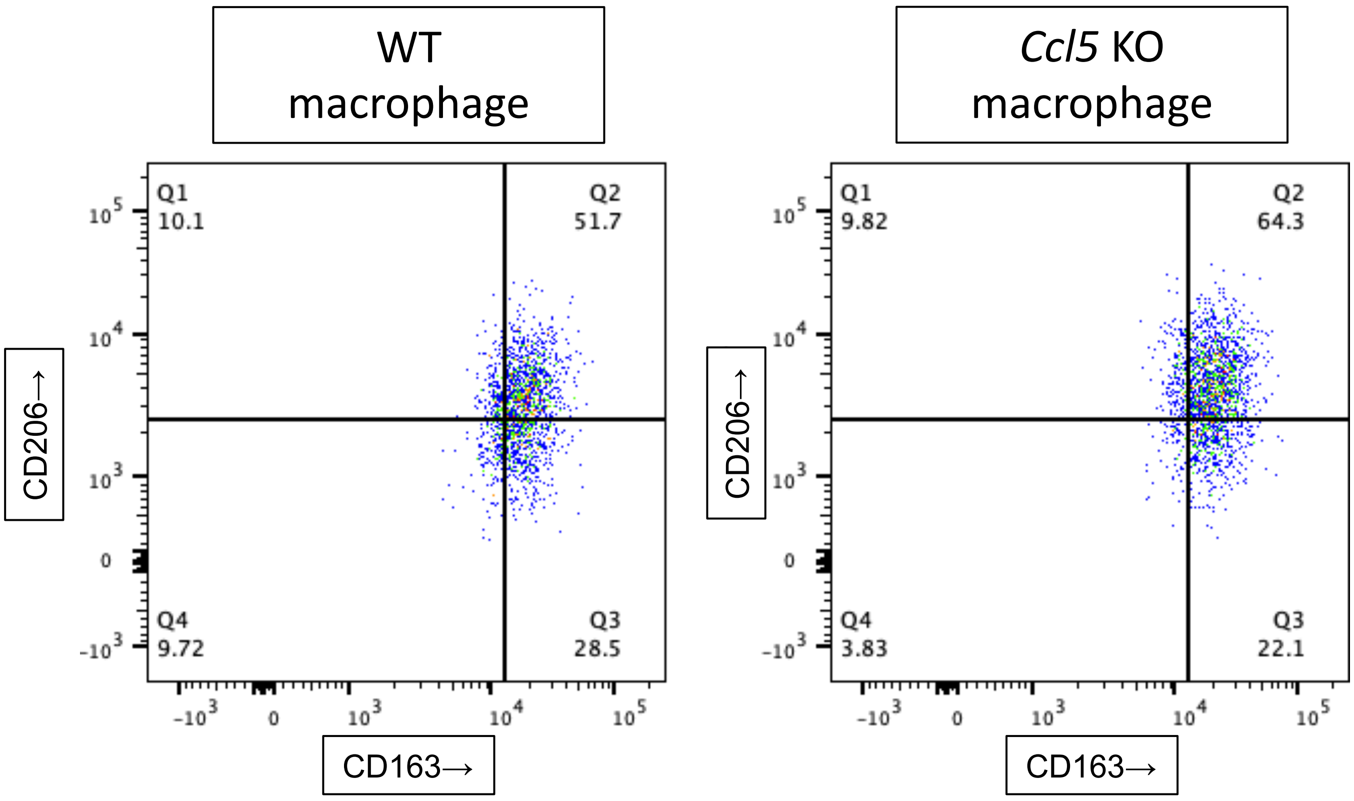

Figure S11. Flowcytometry analysis of polarized M2 macrophages. Representative results of M2 markers macrophages after M2 polarization.

Table S1. Clinical Characteristics of Patients from Human Kidney Biopsy Samples

Age (year old)	Sex	Type of Glomerular Disease	Proteinuria	Urine protein (mg/dl)	Urine creatinine (mg/dl)	Plasma creatinine (mg/dl)	estimated GFR (mL/min/1.73m2)
33	Male	IgAN	2+	104	66	0.88	84.1
56	Male	IgAN	1+	46	71	2	28.6
47	Female	IgAN	2+	64	84	0.75	65.5
65	Male	FSGS	3+	376	41	1.52	37.2
56	Female	FSGS	4+	2944	511	1.6	27
61	Female	FSGS	3+	211	44	1.02	43.5
52	Female	FSGS	2+	160	140	0.63	77.3
41	Male	FSGS	4+	1252	137	3.76	15.9
66	Male	FSGS	3+	309	74	1.02	57.8
43	Female	MCD	3+	301	84	0.82	60.5
49	Male	MCD	4+	2696	305	0.9	71.7
47	Male	MCD	4+	3601	279	0.9	73
18	Female	Lupus	±	18	151	0.35	197.2
51	Male	Lupus	3+	321	79	0.77	84
30	Female	Lupus	3+	364	154	0.97	56.4

Table S2. Predicted Off-Target Sites for CRISPR-Cas9 CCL5 Knockout

Predicted Off-Target Site	Chromosome	Position	Mismatches	Gene Affected
Target (CCL5)	Chr11	-	0	CCL5
Off-target 1	Chr5	115135054	3	Intergenic
Off-target 2	Chr1	194856584	3	Non-coding
Off-target 3	Chr7	144411626	3	Intergenic
Off-target 4	Chr2	147246780	3	Non-coding
Off-target 5	Chr12	116084116	3	Intergenic
Off-target 6	Chr4	151550263	3	Non-coding
Off-target 7	ChrX	7508986	3	Non-coding
Off-target 8	Chr6	34943786	2	Non-coding
Off-target 9	Chr19	42747017	3	Non-coding
Off-target 10	Chr3	28563252	3	Intergenic
Off-target 11	Chr3	104620543	3	Intergenic

Table S3. Antibodies used in this study

Antibodies	Source	Identifier
CCL5	R&D systems	AF478
CD11b	Biolegend	101207
CD163	Biolegend	155309
CD206	Novus Biologicals	NBP1-90020
F4/80	Biolegend	123107
Flag	Sigma-Aldrich	F1804
GAPDH	Sigma-Aldrich	G8795
MHC-II	Biolegend	107607
Synaptopodin	Progen	65194

Table S4. Real-time PCR primers used in this study

Gene Sequence	Source	Identifiers
Ccl5	Thermofisher	Mm 01302428_m1
Ccr1	Thermofisher	Mm 00438260_s1
Ccr3	Thermofisher	Mm 01216172_m1
Ccr5	Thermofisher	Mm 01216171_m1
Gapdh	Thermofisher	Mm99999915_g1
Gpr75	Thermofisher	Mm00558537_s1
Il1b	Thermofisher	Mm00434228_m1
Mrc1	Thermofisher	Mm01329359_m1
