# Supplementary material for: CCL5 paradoxically regulates glomerular injury by skewing macrophage polarization
Source: JCI Insight. 2025 Sep 23;10(21):e173742. doi: 10.1172/jci.insight.173742 (PMC12643500; doi:10.1172/jci.insight.173742)

Uncropped-Unedited
Gel Blots
Fig. 2F

Brightfield Image of the membrane

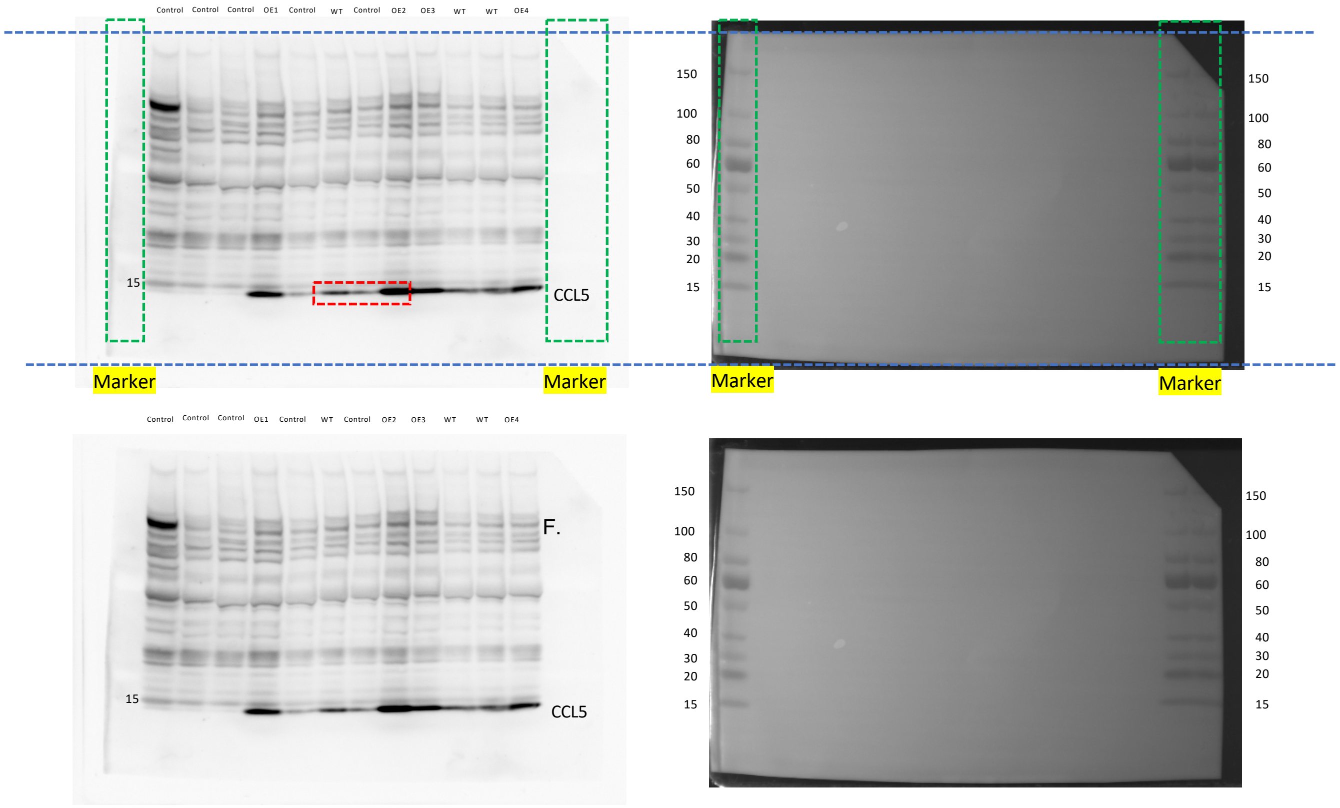

Brightfield Image of the membrane

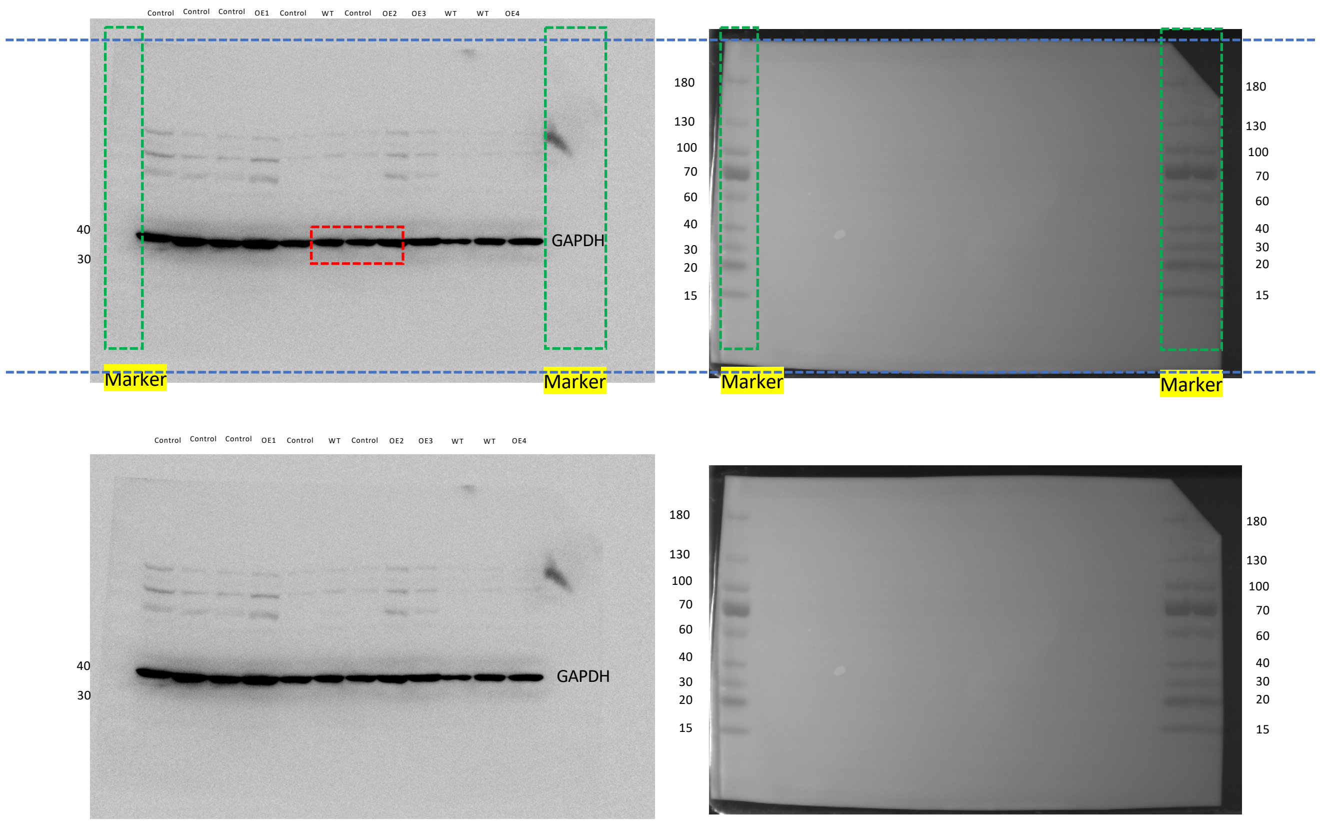

Uncropped-Unedited
Gel Blots
Fig. 3B

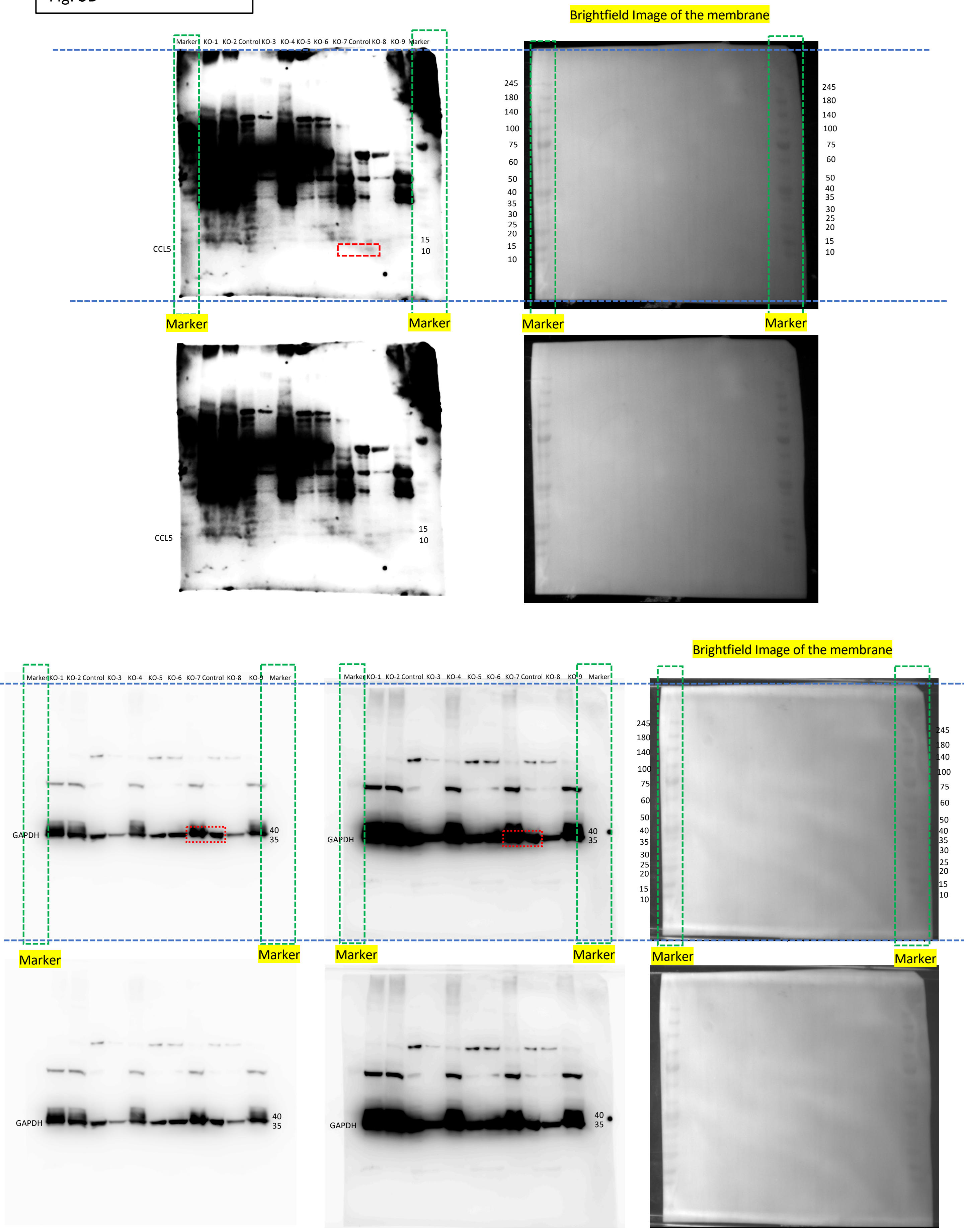

Supplement: Unedited blot and gel images [file jciinsight-10-173742-s108.pdf]
